# Late Maastrichtian pterosaurs from North Africa and mass extinction of Pterosauria at the Cretaceous-Paleogene boundary

**DOI:** 10.1371/journal.pbio.2001663

**Published:** 2018-03-13

**Authors:** Nicholas R. Longrich, David M. Martill, Brian Andres

**Affiliations:** 1 Department of Biology and Biochemistry and Milner Centre for Evolution, University of Bath, Bath, United Kingdom; 2 School of Earth and Environmental Sciences, University of Portsmouth, Portsmouth, United Kingdom; 3 Vertebrate Paleontology Laboratory, The University of Texas at Austin, Austin, Texas, United States of America; Massey University, New Zealand

## Abstract

Pterosaurs were the first vertebrates to evolve powered flight and the largest animals to ever take wing. The pterosaurs persisted for over 150 million years before disappearing at the end of the Cretaceous, but the patterns of and processes driving their extinction remain unclear. Only a single family, Azhdarchidae, is definitively known from the late Maastrichtian, suggesting a gradual decline in diversity in the Late Cretaceous, with the Cretaceous–Paleogene (K-Pg) extinction eliminating a few late-surviving species. However, this apparent pattern may simply reflect poor sampling of fossils. Here, we describe a diverse pterosaur assemblage from the late Maastrichtian of Morocco that includes not only Azhdarchidae but the youngest known Pteranodontidae and Nyctosauridae. With 3 families and at least 7 species present, the assemblage represents the most diverse known Late Cretaceous pterosaur assemblage and dramatically increases the diversity of Maastrichtian pterosaurs. At least 3 families—Pteranodontidae, Nyctosauridae, and Azhdarchidae—persisted into the late Maastrichtian. Late Maastrichtian pterosaurs show increased niche occupation relative to earlier, Santonian-Campanian faunas and successfully outcompeted birds at large sizes. These patterns suggest an abrupt mass extinction of pterosaurs at the K-Pg boundary.

## Author summary

Pterosaurs were winged cousins of the dinosaurs and lived from around 200 million years ago to 66 million years ago, when the last pterosaurs disappeared during the Cretaceous-Paleogene extinction that wiped out the dinosaurs. The pterosaurs are thought to have declined in diversity before their final extinction, suggesting that gradual processes played a major role in their demise. However, pterosaur fossils are very rare, and thus, it is unclear whether pterosaurs were really low in diversity at this time or whether these patterns merely result from a paucity of fossils. We describe new pterosaur fossils from the end of the Cretaceous in Morocco, including as many as 7 species. They represent 3 different families and show a large range of variation in size and skeletal proportions, suggesting that they occupied a wide range of ecological niches.

## Introduction

Pterosaurs first appear in the fossil record in the Late Triassic [1–3], tens of millions of years before birds took wing [4]. Like birds, pterosaurs were archosaurs capable of powered flight; unlike birds, they flew on membraneous wings, supported by an elongate fourth digit, and walked or climbed on all fours [2,5,6]. After appearing in the Triassic, pterosaurs radiated in the Jurassic [2,7–9], followed by a second radiation of advanced, short-tailed pterodactyloid pterosaurs in the Early Cretaceous [2,7–12]. By the mid-Cretaceous, pterosaurs had evolved aerial insectivores, carnivores, piscivores, durophages, and filter feeders [2,5,6] and exploited habitats from forests [13], lakes [12], coastal plains [14], and deserts [15,16] to shallow seas [2] and the open ocean [17]. The smallest pterosaurs had a wingspan of 50 cm or less [13,18]; the largest had wingspans of 10–11 m and weighed 200–250 kg [19], making them the largest flying animals ever to evolve.

How and why this long-lived, diverse clade became extinct remains unclear. Pterosaur diversity declined in the mid-Cretaceous [5,7,8], but at least 4 clades—Azhdarchidae [2], Nyctosauridae [2], Pteranodontidae [2], and Tapejaridae [16]—and perhaps a fifth lineage, represented by the enigmatic *Piksi barbarulna* [20], persist into the final 25 million years of the Cretaceous, before seeming to gradually disappear towards the end of the Cretaceous. Only a single family, Azhdarchidae, is definitively known from the Maastrichtian [2,5,21]. The youngest pteranodontids are early Campanian in age [2,18]. Nyctosaurids persisted until the Campanian at least, but the youngest nyctosaurid, “*Nyctosaurus” lamegoi*, lacks formation-level provenance data [22] and may be Campanian or Maastrichtian [22], making the timing of extinction uncertain [22]. When Tapejaridae became extinct is also unclear. The Santonian *Bakonydraco galaczi* [23] has been interpreted as a tapejarid [24], extending tapejarids into the middle Late Cretaceous [23]; the tapejarid *Caiuajara dobruskii* [16] could be as young as Campanian or as old as Turonian [16]. The enigmatic *Piksi* is late Campanian [25] in age.

Along with a decline in number of families towards the Cretaceous–Paleogene (K-Pg) boundary, pterosaurs’ species richness [8] and morphological disparity [9] are thought to decrease prior to their ultimate extinction. These patterns have been interpreted as showing a gradual decline in pterosaur diversity in the late Cretaceous [26]. If so, the K-Pg extinction may have been the final blow to a group whose extinction had long been underway and was perhaps inevitable [6].

However, the pterosaur record is highly incomplete, raising the possibility that sampling artifacts drive these patterns. Sampling effects can cause abrupt extinctions to appear gradual [27], an artifact known as the Signor-Lipps Effect: the last fossil of a lineage appears some point before its extinction. When this artifact affects many species at once, it can cause catastrophic extinctions to appear drawn out [27]. The Signor-Lipps Effect should be strongest for groups with a highly incomplete record. Pterosaurs represent an extreme case, because their thin-walled, hollow bones have low preservation potential [2]. The gradual disappearance of pterosaur families, therefore, could be a sampling artifact. Similarly, observed declines in diversity [8] and disparity [9] could be driven by changes in the quality of the fossil record [8,9], given that the number of formations preserving pterosaurs declines from the Campanian to the Maastrichtian [8]. The completeness of pterosaur fossils also decreases [28] such that the available fossils may provide less information on species richness and disparity. Furthermore, the pterosaur record is dominated by Lagerstätten [8,11,28,29]—localities with exceptional preservation. Pterosaur diversity is concentrated in these Lagerstätten, notably the Solnhofen [2], Yixian, Jiufotang [11], Romualdo [2,30,31], Crato [10], Cambridge Greensand [32,33], and Niobrara [34,35] formations [2,5,10], such that a dozen such formations account for around half of known diversity [28]. However, no Lagerstätten are known from the final 15 million years of the Cretaceous. Finally, end Cretaceous pterosaurs are primarily known from terrestrial horizons, with few occurrences in marine settings [22,36,37], which may provide an incomplete record of marine lineages.

**Fig 1:**
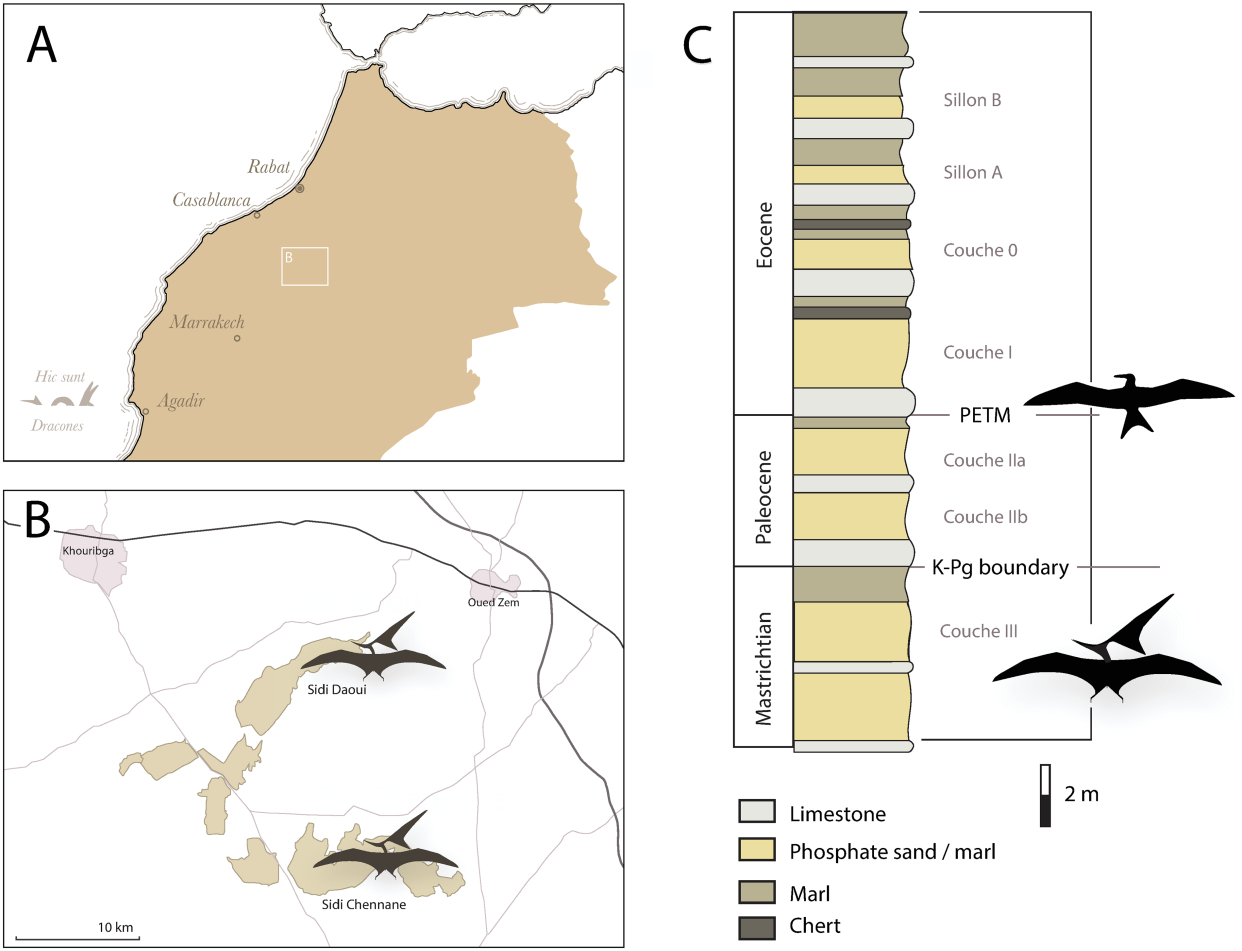
A) Map showing the location of the phosphate mines in Morocco, (B) map showing Sidi Daoui and Sidi Chennane mines, and (C) stratigraphic column for the phosphates of the Sidi Daoui area (after [40]). Abbreviations: PETM, Paleocene-Eocene Thermal Maximum.

These processes—the Signor-Lipps Effect and changes in the quality of the fossil record—may drive the apparent decline in pterosaurs. If so, improved sampling should reveal additional diversity and disparity in the latest Cretaceous. To test this hypothesis, we studied a remarkable new collection of pterosaurs from the late Maastrichtian [38,39] phosphates of the Khouribga Plateau in Morocco (Fig 1A), North Africa [40]. Here we provide a preliminary description of this fauna and explore its implications for pterosaur extinction.

## Results

### Geological setting

The fossils described here come from the upper Maastrichtian phosphates of the Ouled Abdoun Basin, in northern Morocco. Commercial exploitation of the phosphates has uncovered large numbers of marine vertebrates [41] from the Maastrichtian and early Paleogene [41]. The Cretaceous fauna includes an extraordinary diversity of marine reptiles, including mosasaurs, plesiosaurs, and turtles [41,42], abundant and diverse bony fish [42], sharks [38], and pterosaurs [43], as well as rare dinosaurs [44,45]. Preliminary studies indicate that the fauna is the most diverse and abundant known Maastrichtian marine vertebrate assemblage.

These beds have not been formally assigned to a formation; instead, a series of beds or “Couches” are informally designated for the purposes of the mining industry (Fig 1C). Couche III is Late Cretaceous in age, and Couche I and Couche II are early Paleogene. Vertebrate biostratigraphy places Couche III in the upper Maastrichtian [38], and carbon and oxygen isotope chemostratigraphy constrain Couche III to the latest Maastrichtian, within approximately 1 Ma of the K-Pg boundary [39]. The fauna therefore provides a picture of a marine ecosystem just before the K-Pg extinction.

Until now, the pterosaur record from the assemblage comprised a single specimen, the holotype of the azhdarchid *Phosphatodraco mauritanicus* [43]. Over the past 3 years, we have worked with the local fossil industry to assemble a collection of pterosaurs that includes over 200 specimens, ranging from isolated bones to partial skeletons. This collection is currently the largest and most diverse collection of Maastrichtian pterosaurs in the world. Fossils primarily occur as disarticulated bones, but associated bones and, rarely, partial skeletons have also been recovered. Most come from dense, laterally extensive bonebeds in the middle of Couche III at Sidi Daoui, and a minority come from Couche III at Sidi Chennane (Fig 1B). Several specimens originate in a lower layer, about 2 m below Couche III, which is characterized by a fine, pale grey matrix and white bone. The age of these fossils is unknown, and thus, they are not described here.

### Systematic paleontology

The fauna comprises a minimum of 7 species, including 1 species of Pteranodontidae, 3 species of Nyctosauridae, and 3 species of Azhdarchidae.

Archosauria Cope, 1869

Pterosauria Kaup, 1834

Pterodactyloidea Plieninger, 1901

Ornithocheiroidea *sensu* Kellner, 2003 [46]

Pteranodontoidea *sensu* Kellner, 2003 [46]

Pteranodontia *sensu* Unwin, 2003 [47]

Pteranodontidae Marsh, 1876

### *Tethydraco regalis* gen. et sp. nov.

urn:lsid:zoobank.org:act:E5036E72-9C55-4BD8-9315-552F572781F

**Etymology**. The genus derives from Tethys, in reference to the Tethys Sea, and the Latin *draco*, “dragon.” The species name is derived from the Latin *regalis*, “royal.”

**Fig 2:**
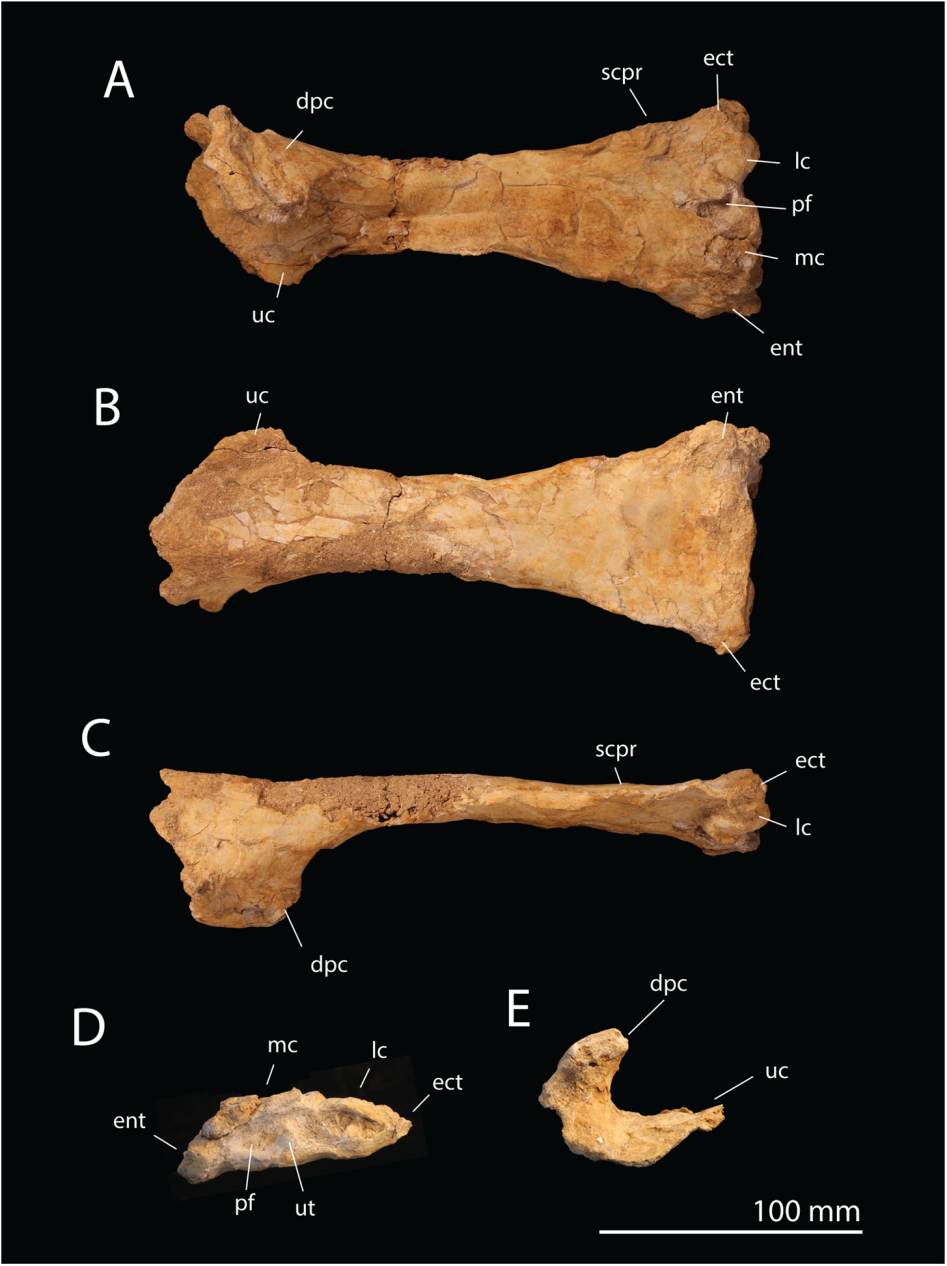
*T*. *regalis* FSAC-OB 1, holotype left humerus. In (A), ventral view, (B) dorsal view, (C) anterior view, (D) distal view, and (E) proximal view. Abbreviations: dpc, deltopectoral crest; ect, ectepicondyle; ent, entepicondyle; lc, lateral condyle; mc, medial condyle; pf, pneumatic fossa/foramen; scpr, supracondylar process; uc, ulnar crest; ut, ulnar tubercle.

**Fig 3:**
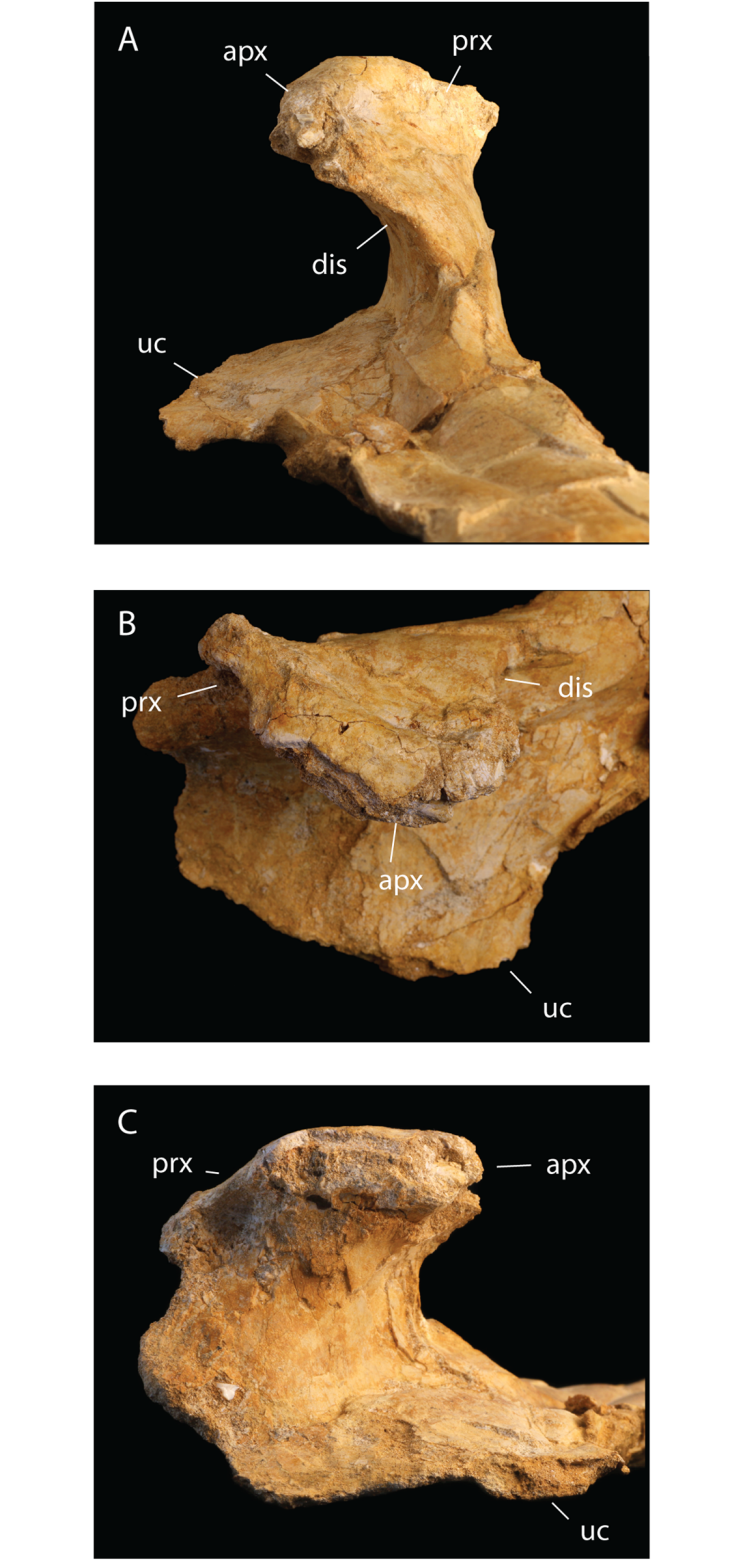
*T*. *regalis* FSAC-OB 1, deltopectoral crest. In (A), distal view, (B) apical view, and (C) proximal view. Abbreviations: apx, apex of deltopectoral crest; dis, distal margin of deltopectoral crest; prx, proximal margin of deltopectoral crest; uc, ulnar crest.

**Holotype**. FSAC-OB 1, left humerus (Figs 2 and 3).

**Horizon and locality**. Middle Couche III; Sidi Daoui, Khouribga Province, Morocco.

**Fig 4:**
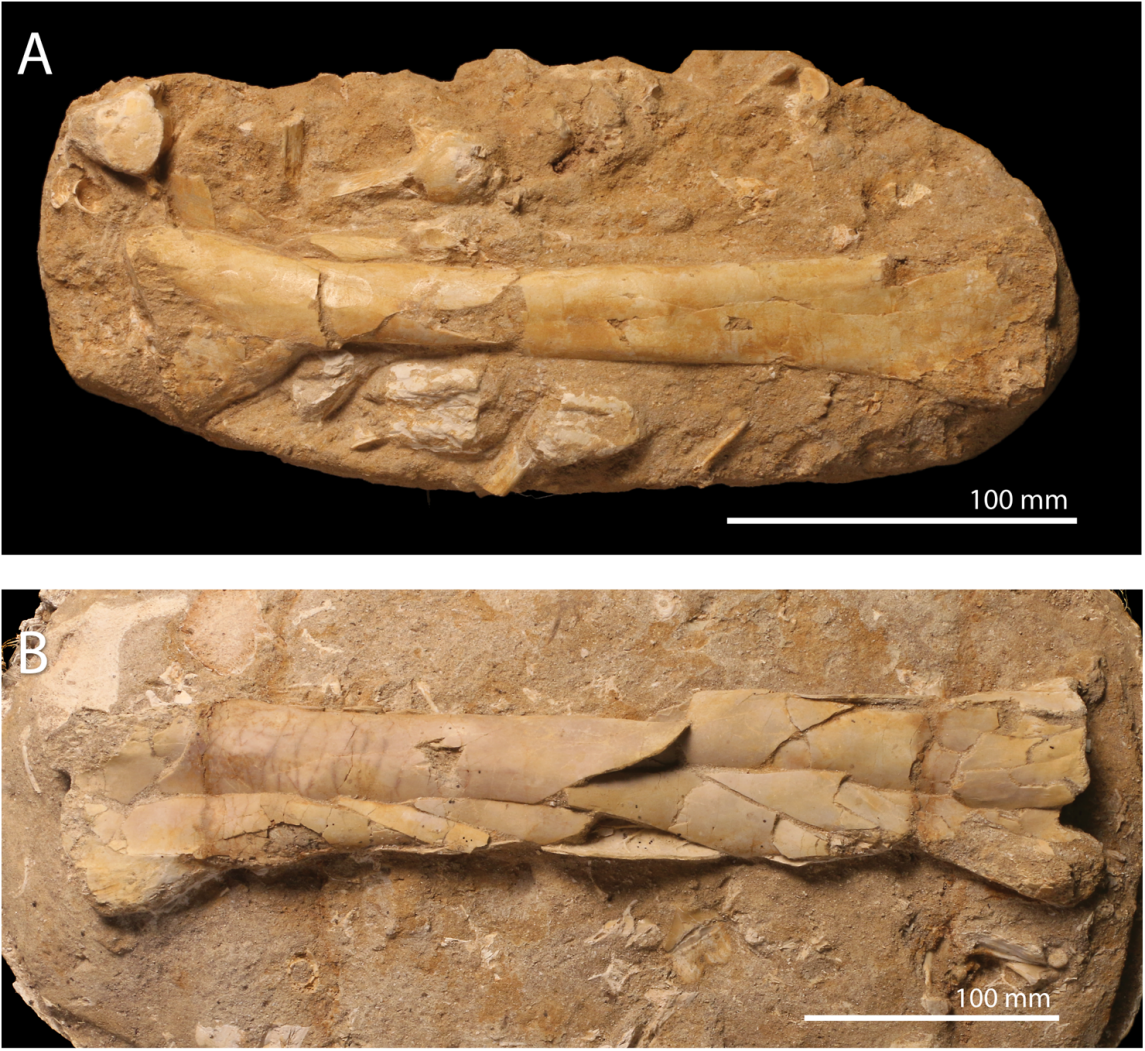
cf. *T*. *regalis* FSAC-OB 199 and 200, ulnae. (A) Right ulna FSAC-OB 199 in posterior view; (B) right ulna FSAC-OB 200 in posterior view.

**Fig 5:**
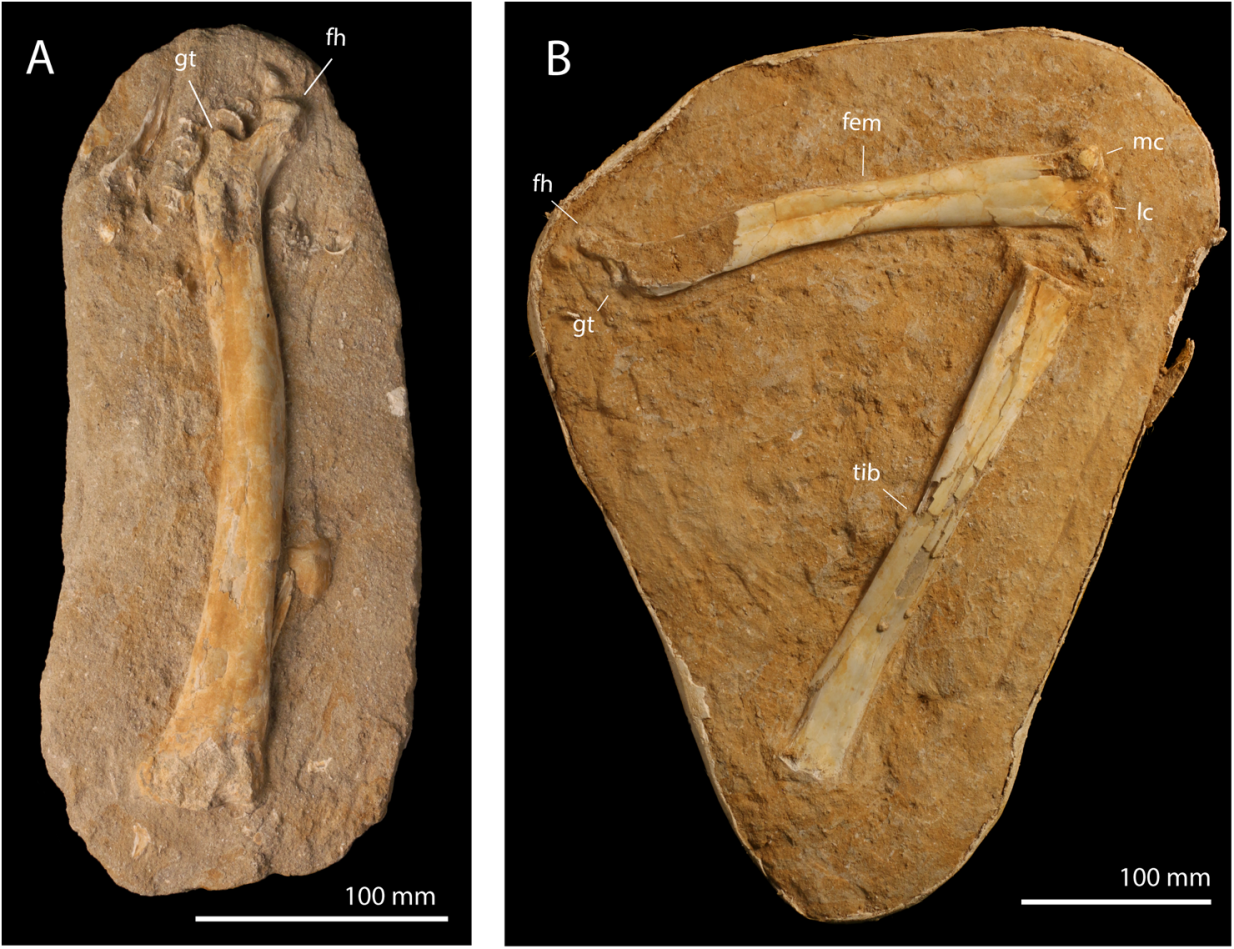
cf. *T*. *regalis* FSAC-OB 201 and 201 hindlimb elements. (A) Right femur FSAC-OB 201 in anterior view; (B) left femur and tibia FSAC-OB 202 in posterior view. Abbreviations: fem, femur; fh, femoral head; gt, greater trochanter; lc, lateral condyle; mc, medial condyle; tib, tibia.

**Referred material**. FSAC-OB 199 ulna (Fig 4A), FSAC-OB 200 ulna (Fig 4B); FSAC-OB 201, femur (Fig 5A), and FSAC-OB 202 femur and tibia (Fig 5B).

**Diagnosis**. Pteranodontid with a deltopectoral crest that is small and proximally placed, terminating just past the end of the ulnar crest; a very broad, triangular distal end of the humerus; an ectepicondyle with a prominent dorsal projection; and an entepicondyle that is enlarged and proximally extended. The ulna is proportionately short and broad, with a massively expanded proximal end.

**Description**. The humerus measures 231 mm long but is missing the head. The length of the humerus implies a wingspan of approximately 5 m, making *Tethydraco* comparable to other pteranodontids in size.

The shaft is broad proximally, narrows past the deltopectoral crest, and distally becomes a broadly expanded, triangular structure. As in *Pteranodon* [34] and ornithocheirids [30], the deltopectoral crest is trapezoidal in shape; the base is broad, but it tapers toward the apex. However, the deltopectoral crest is smaller and more proximally placed than in *Pteranodon* (S1 Fig), with the base of the crest extending slightly past the ulnar crest and the apex terminating above the ulnar crest. This feature is an autapomorphy that distinguishes *Tethydraco* from other Pteranodontidae.

The deltopectoral crest is massively constructed. The proximal and distal margins of the crest are thin, but then the deltopectoral crest becomes thicker towards the middle, with a broad, massive pillar of bone extending along the ventral surface of the crest from its base to its apex. The apex of the deltopectoral crest is also broadly expanded. This thickened crest is absent in Azhdarchidae or Nyctosauridae but is seen in *Pteranodon* [34] and Ornithocheirae [30]. The deltopectoral crest is strongly curled such that its tip actually hooks backward. This feature is not seen in nyctosaurids or azhdarchids. This feature may be present in *Pteranodon* but is difficult to assess given the crushing of the material, but a strong posterior curling of the deltopectoral crest is seen in ornithocheirids [30], if not to the degree seen in *Tethydraco*.

The deltopectoral crest differs from that of Azhdarchidae and Nyctosauridae but resembles that of other Pteranodontidae [34] and ornithocheirids [30] in being warped. The entire deltopectoral crest curls posteriorly, but this curvature is more strongly developed distally than proximally, such that the deltopectoral crest is twisted about its long axis: at its tip, the dorsal surface of the crest is angled to face distally, and the apex of the deltopectoral crest is rotated so that its long axis lies at an angle of 45° to the humerus, rather than lying parallel to the long axis as in other Late Cretaceous families such as Nyctosauridae and Azhdarchidae.

The distal half of the humeral shaft is expanded and triangular and closely resembles *Pteranodon* (S2 Fig). This derived feature of pteranodontids [48] is shared with nyctosaurids (see below) but is absent in azhdarchoids, in which the middle of the shaft is relatively cylindrical, and the distal one-third of the humeral shaft is strongly expanded [49]. The distal expansion is extreme, with the distal width being more than twice the width of the shaft at its narrowest point and perhaps as much as one-third the total humerus length, giving the end of the humerus a paddle-like shape. Neither *Pteranodon* nor any other pterosaur has a similarly extreme shape, and it appears to be an autapomorphy of *Tethydraco*.

The lateral condyle resembles that of *Pteranodon* [48]; the medial condyle is damaged. A large oval pneumatic foramen appears to lie between the distal condyles as in other pterosaurs, including *Pteranodon* [48].

The ectepicondyle is prominent and projects strongly laterally. This strong lateral projection is a derived feature shared with *Pteranodon* [48], but it is better developed in *Tethydraco*, an autapomorphy that helps to diagnose *Tethydraco*.

The supracondylar process is proximally positioned, lying about one-quarter of the way from the end of the humerus. The crest is long and narrow but relatively low, unlike the prominent flange seen in nyctosaurids.

The entepicondyle projects medially and distally. The strong distal projection of the entepicondyle is a derived feature shared with both *Pteranodon* and the Nyctosauridae, although it is better developed in nyctosaurids than pteranodontids. The broad medial projection of the entepicondyle is shared with Pteranodontidae to the exclusion of other pterosaurs and represents a pteranodontid feature.

The distal end of the humerus is strongly compressed. Although this condition may be exaggerated by crushing, the rest of the humerus is relatively 3-dimensional, suggesting that the end of the humerus was dorsoventrally flattened in life.

The humerus is subtriangular in distal view, with a relatively straight dorsal margin, an ornithocheiroid feature, rather than a D-shaped one as would be expected for an azhdarchid, in which the dorsal margin is strongly convex. In distal view, there is a deep olecranon fossa with a prominent ulnar tubercle. There is a pneumatic foramen below the medial condyle. On the posterior surface of the shaft is a shallow trough for the passage of the triceps brachii.

Two pteranodontid ulnae are known from the phosphates. FSAC-OB 200 (Fig 4A) is smaller but better preserved than FSAC-OB 200 (Fig 4B); it compares well with *Pteranodon* in shape (S3 Fig). The proximal end of the ulna in FSAC-OB 199 is extremely broad, about twice the minimum diameter of the shaft. This feature may be autapomorphic and presumably corresponds with the strong distal expansion of the humerus. The shaft is relatively short and robust as in other Pteranodontidae [34,48,50] and gradually expands in diameter distally. Distal expansion of the shaft is seen in other pteranodontids [34,48,50] but is better developed in *Tethydraco*, such that there is no clear demarcation between the shaft and the distal end of the ulna as seen in other taxa.

The referred femora, FSAC-OB 201 and 202, resemble those of *Pteranodon* (S4 Fig) [34] and are short and robust compared to azhdarchoids. The femoral head is proximally directed as in other pterosaurs and is well developed and ball-like, in contrast to nyctosaurids, in which the head is reduced [35]. A narrow neck connects the femoral head to the shaft; by contrast, the neck is distally expanded and massive in nyctosaurids [35]. The greater trochanter is large and developed as a prominent proximal prong set off from the femoral head by a deep notch, as in *Pteranodon*. The greater trochanter is reduced in nyctosaurids [35]. The shaft is distinctly bowed medially as in other Pteranodontidae and Nyctosauridae. The shaft is relatively constant in diameter along its length, in contrast to Nyctosauridae, in which the distal half of the shaft is expanded [35].

The tibia, FSAC-OB 202, is proportionately short and robust relative to that of *Pteranodon*.

**Comments**. *Tethydraco* represents both the first report of a pteranodontid from the Maastrichtian and the first known from Africa. Numerous features allow referral to Pteranodontidae. The humerus is distinguished from nyctosaurids by the deltopectoral crest, which lacks a hatchet-shaped distal expansion, and from both nyctosaurids and azhdarchids by the massive construction, curling, and warping of the deltopectoral crest. The humeral shaft differs from Azhdarchidae in that the distal half is strongly expanded. The humerus differs from both nyctosaurids and azhdarchids in having a prominent, laterally projecting ectepicondyle and a large, triangular, medially projecting entepicondyle; in these features, it resembles *Pteranodon*. The ulna resembles pteranodontids in being robust, with a distally expanded shaft. The femur is characteristic of pteranodontids in having a combination of a bowed shaft, a derived character of the Pteranodontidae and Nyctosauridae, with a well-developed femoral head, a prominent greater trochanter, and an unexpanded distal femoral shaft, plesiomorphies absent in nyctosaurids.

Despite the similarities, *Tethydraco* differs from *Pteranodon* in having a smaller, proximally positioned deltopectoral crest and a more prominent ectepicondyle. The shape is different as well, with the shaft being more elongate and more strongly expanded distally. The ulna has a strong expansion of the proximal end and distal expansion of the shaft. These features distinguish *Tethydraco* from *Pteranodon*. The femur is indistinguishable from *Pteranodon*.

Referral of the ulna to *Tethydraco* is made on the basis of size and the strong transverse expansion of the elbow joint, which corresponds to the expanded end of the humerus seen in the holotype. Referral of the femur is more tentative, being made on the basis of size and provenance. It is entirely possible that more than one pteranodontid existed in the assemblage but in the absence of any evidence for more than one species, it is provisionally referred to *Tethydraco*. Associated material is required to test this referral.

Nyctosauridae Nicholson and Lyddekker 1889

### *Alcione elainus* gen. et sp. nov.

urn:lsid:zoobank.org:act:928D37AF-8C3E-4168-BF88-6531B3EC520B

**Etymology**. The genus name is from Alcyone of Greek mythology, who was turned into a seabird, and the species name is from the Greek *elaino*, “to stray or wander.”

**Diagnosis**. Small nyctosaur. The scapula and coracoid are subequal in length. The humerus is short and robust, with a strongly expanded proximal end; proximal pneumatic fossa and foramen are absent. The deltopectoral crest is positioned proximally, close to the head of humerus, with strong constriction at midlength producing an exaggerated hatchet shape and an acutely pointed distal prong. It has a very large, proximally positioned supracondylar process. The entepicondyle is hypertrophied and distally projecting. The antebrachium and metacarpal IV are short and robust. The femur is short and robust.

**Fig 6:**
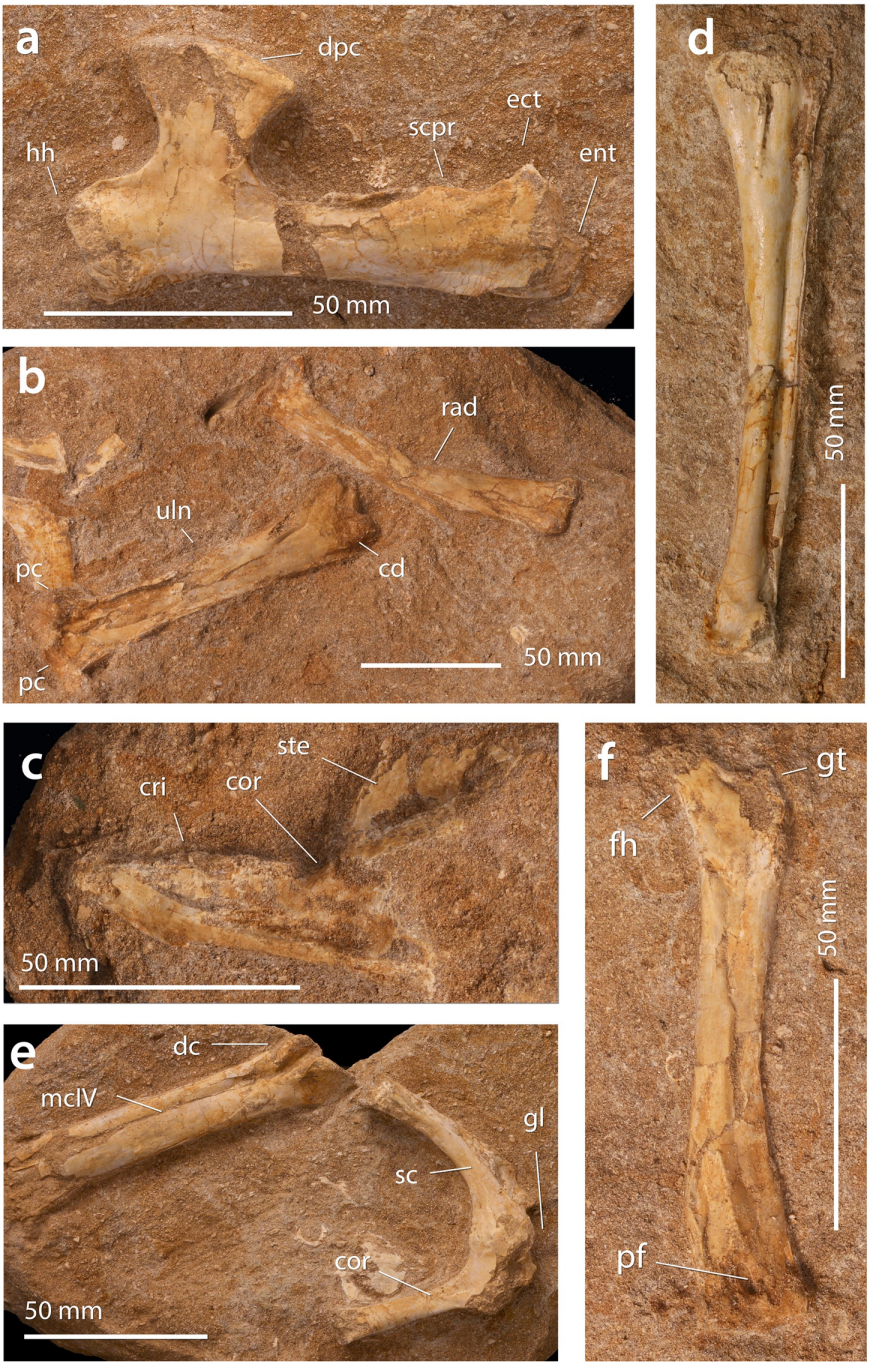
*A*. *elainus* FSAC-OB 2, holotype partial skeleton and FSAC-OB 217, metacarpal IV. (A) Holotype right humerus in anterior view, (B) holotype right ulna and radius in anterior view, respectively, (C) holotype sternum in left lateral view, (D) referred metacarpal IV, (E) holotype, distal end of left metacarpal IV and left scapulocoracoid, and (F) holotype right femur in posterior view. Abbreviations: co, coracoid; cr, cristospine; dc, distal condyle; dpc, deltopectoral crest; ect, ectepicondyle; fh, femoral head; gl, glenoid; gt, greater trochanter; hh, humeral head; hum, humerus; mcIV, metacarpal IV, pc, proximal cotyle; pf, pneumatic foramen; rad, radius; scpr, supracondylar process; ste, sternum; uln, ulna.

**Holotype**. FSAC-OB 2 (Fig 6), partial skeleton including humerus, sternum, scapulocoracoid, and femur.

**Type locality**. Middle Couche III; Sidi Daoui, Khouribga Province, Morocco.

**Fig 7:**
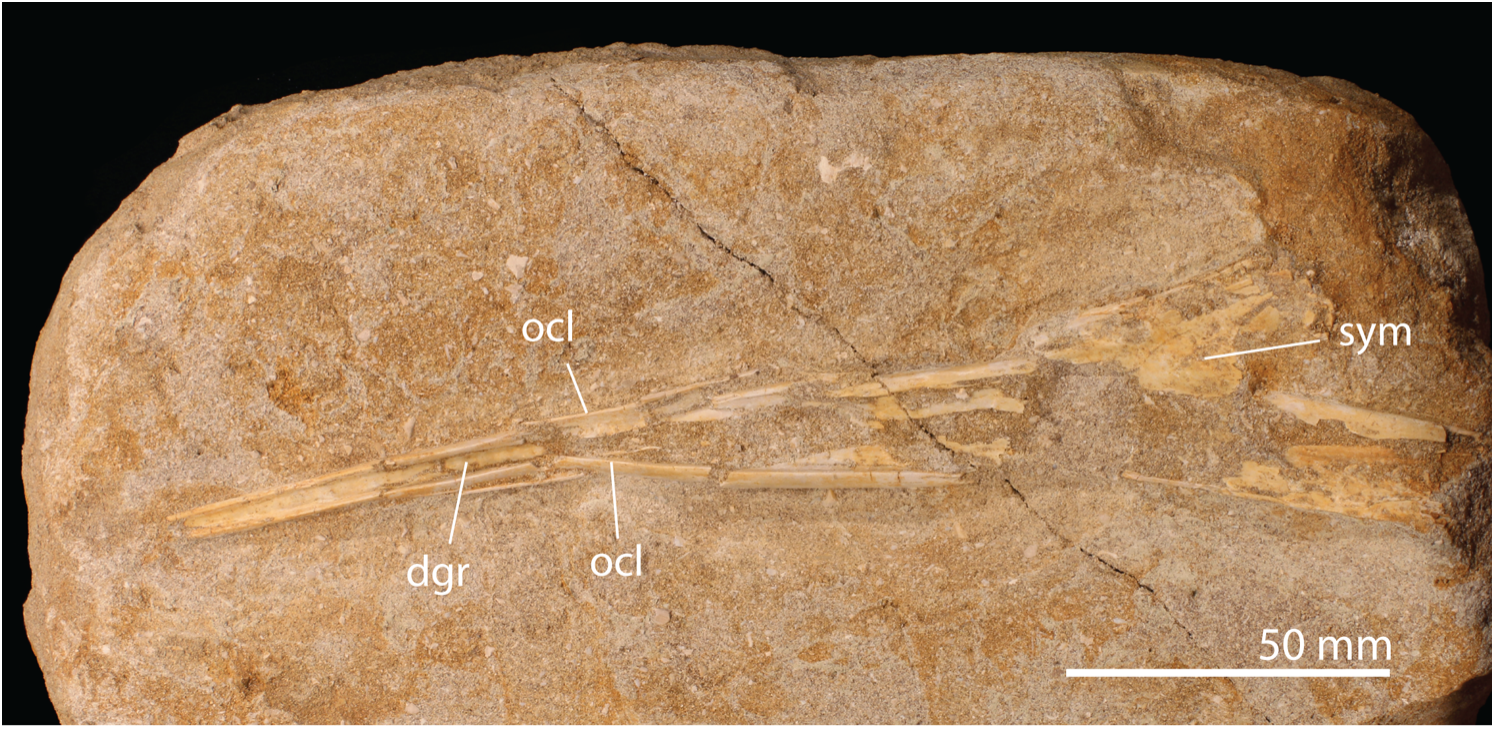
*A*. *elainus* FSAC-OB 156 mandible. Abbreviations: dgr, dorsal groove; ocl, occlusal ridge; sym, symphysis.

**Fig 8:**
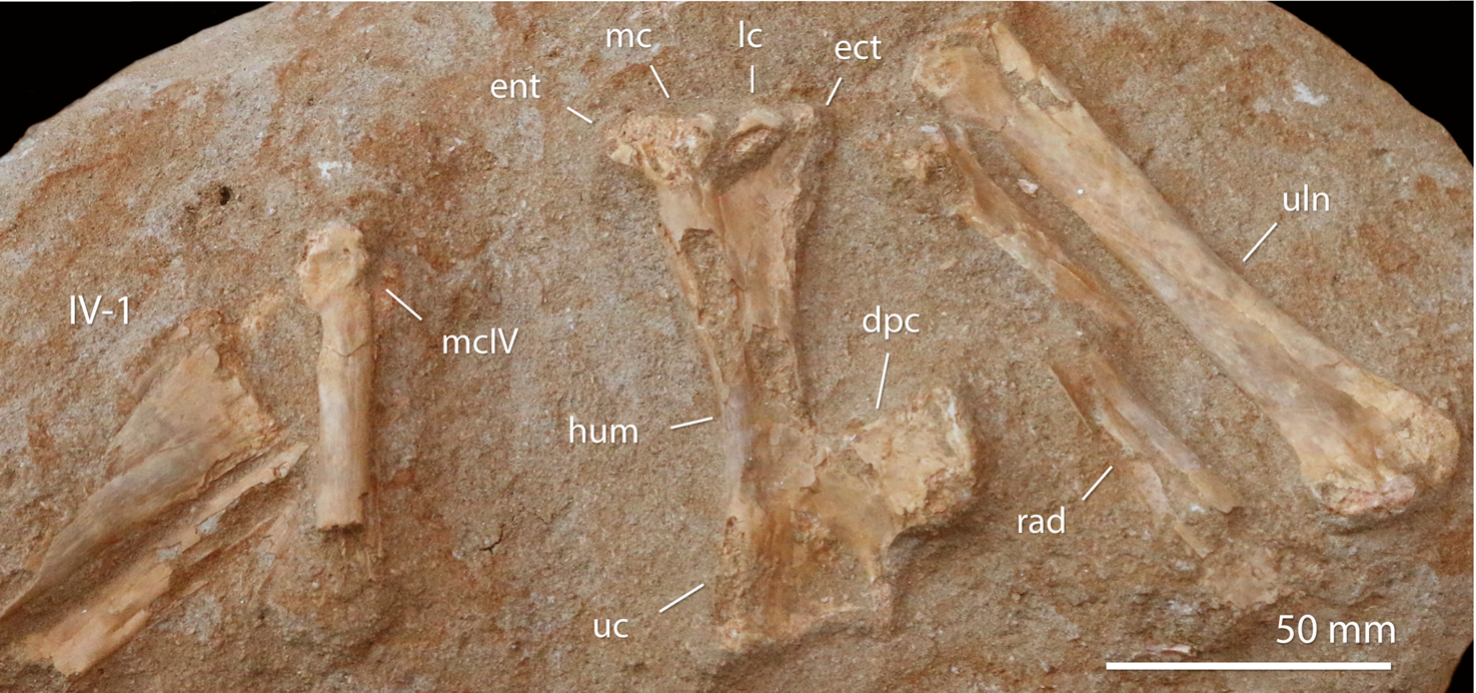
*Alcione* FSAC-OB 4, partial right wing. Humerus in ventral view, ulna and radius in posterior view, and metacarpal IV and phalanx IV-1 in ventral view. Abbreviations: dpc, deltopectoral crest; ect, ectepicondyle; ent, entepicondyle; hum, humerus; lc, lateral condyle; mc, medial condyle; mcIV, metacarpal IV; IV-1, first phalanx of digit IV; rad, radius; uln, ulna.

**Referred material**. FSAC-OB 217, metacarpal IV (Fig 6D); FSAC-OB 156 mandible (Fig 7); FSAC-OB 4, partial wing including humerus, radius, ulna, parts of metacarpal IV and phalanx IV-1 (Fig 8), and additional postcrania including humeri, ulnae, radii, scapulocoracoids, and synsacra (S1 Table).

**Description**. The referred mandible (Fig 7) resembles other nyctosaurids, with the fused dentaries forming a long, slender, Y-shaped element in dorsal view. Teeth are absent; the occlusal margins of the beak have sharp edges for a rhamphotheca. In the anterior third of the jaw, the occlusal edges are nearly parallel, giving the beak a needle-like shape. Near the middle of the jaw, the occlusal edges diverge, and a broad, shallow trough, triangular in shape, is developed between them.

The scapulocoracoid in the holotype is fused (Fig 6E), suggesting somatic maturity [51]. The bone is boomerang shaped as in other nyctosaurids [35] and, to a lesser degree, in *Pteranodon* [34]. The scapula is straight with a robust acromion process. The coracoid is gently curved and bears a triangular flange below the glenoid.

The sternum (Fig 6C) bears a prominent, triangular cristospina, as in other nyctosaurids [35] and pteranodontids [34]. An oval sternocoracoid articulation is preserved and appears to have had a constriction posterior to it.

**Fig 9:**
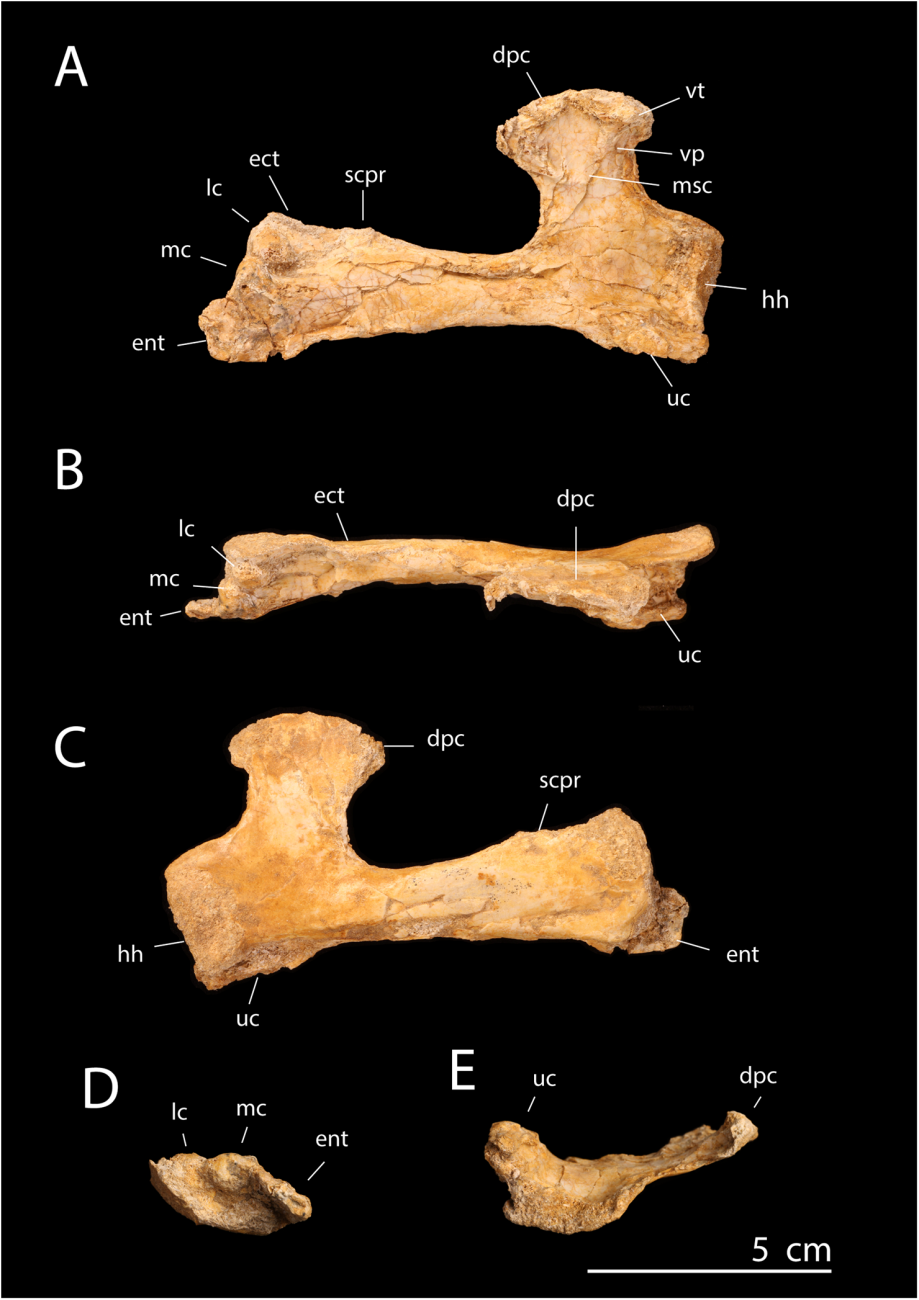
*Alcione* FSAC-OB 5, right humerus. In (A), ventral view, (B) anterior view, (C) dorsal view; (D) distal view, and (E) proximal view. Abbreviations: dpc, deltopectoral crest; ect, ectepicondyle; ent, entepicondyle; hh, humeral head; lc, lateral condyle; mc, medial condyle; msc, muscle scar; uc, ulnar crest, vp, ventral ridge; vt, ventral tubercle.

The humerus (Figs 6A, 8 and 9) has a straight shaft with a sharp inflection proximal to the deltopectoral crest, deflecting the humeral head dorsally. The head’s posteroventral face bears a deep fossa just proximal to the deltopectoral crest but lacks a pneumatic foramen. The ulnar crest is a rounded rectangular flange projecting posteriorly.

The deltopectoral crest is oriented obliquely with respect to the humeral shaft but lacks the curling and twist seen in pteranodontids [34] and Ornithocheirae [30] or the thickening of the deltopectoral crest and its apex seen in those taxa. Instead, it is a thin, flat plate that projects ventrally from the humeral shaft. Its lateral surface is gently concave, and its medial surface (Fig 9A) bears a ridge running from base to apex, the ventral pillar. Running from the ventral pillar to the anterodistal edge of the deltopectoral crest is an oblique ridge, probably a muscle scar.

The deltopectoral crest has the hatchet shape characterizing nyctosaurids [35,52–55], narrowing at midlength and expanding again distally. The deltopectoral crest is strongly constricted in *Alcione*, with its width at the narrowest point being about 65% of its distal width. A similar constriction is seen in *Nyctosaurus nanus* YPM 1182 [53] and *Nyctosaurus* FMNH P 25026 [35] but is more weakly developed in *Nyctosaurus “bonneri”* SM 11311 [52], *Muzquizopteryx coahuilensis* [54,55], and “*N*.” *lamegoi* [22]. The distal prong of the apex is prominent and acutely pointed, and the anterior margin of the deltopectoral crest is strongly concave, as in *N*. *nanus* [53] and FMNH P 25026 [35]. The proximal prong is a prominent, bluntly rounded process.

The supracondylar process of the ectepicondyle is shifted well proximal to the distal condyles of the humerus and is developed as a large flange, a derived feature of nyctosaurids. The entepicondyle is developed as a prominent posteroventrally projecting flange, as in pteranodontids, but differs in projecting distally well past the condyles, an autapomorphy of *Alcione*. The humerus’s distal end bears a small groove between the condyles that terminates in a pneumatic foramen. The distal condyles are well ossified in most specimens, implying that they are near or at maturity [51].

The antebrachium is short and robust (Figs 6B and 8), being 128% the length of the humerus in the type; the antebrachium of *Nyctosaurus* is 170%–184% the length of the humerus.

The femur (Fig 6F) resembles other nyctosaurids [35,55] and, to a lesser degree, pteranodontids. The femoral head is highly reduced, but the neck is expanded distally where it meets the shaft. The femoral shaft is strongly bowed medially. A small pneumatic foramen is present between the femoral neck and a distinct greater trochanter, with a larger foramen positioned between the distal condyles on the posterior surface. The femoral shaft is distally expanded and lacks distinct epicondyles, as in *Nyctosaurus* [35,55].

**Comments**. *Alcione* is the first nyctosaurid assignable to the late Maastrichtian and the first nyctosaurid from Africa. It is referred to Nyctosauridae based on the hatchet-shaped deltopectoral crest with a distinct ventral ridge and the lack of a warped deltopectoral crest. It differs from other nyctosaurids in numerous characters. The proximal end of the humerus is broader, the distal prong of the deltopectoral crest is acutely pointed, the antebrachium and wing metacarpal are much shorter, and the femur is unusually short and robust. All of these features show that it is a new taxon.

Within *Alcione*, there is a high degree of variation, especially in the shape of the deltopectoral crest (S5 Fig). The deltopectoral crest ranges from narrow, with a straight apex (Fig 7A), to broader, with a more convex apex (Fig 8). The significance of this variation is unclear. Most individuals are similar in size and, based on bone texture, appear to be mature; this suggests that this shape variation is not ontogenetic. Intraspecific variation is another possibility. A third possibility is that the current sample includes 2 or more species. This might not be surprising given that in many extant marine bird faunas, a given genus may include 3 or more co-occurring species; examples include the albatross *Phoebastria*, the frigatebird *Fregata*, the booby *Sula*, the gull *Larus*, and the tern *Sterna* [56], in which multiple species have overlapping ranges. Additional, associated remains are needed to test these hypotheses.

The abbreviated distal wing elements in *Alcione* indicate a specialized flight style. The short, robust proportions suggest reduced wingspan and increased wing loading, implying distinct flight mechanics and an ecological shift. Short wings would increase lift-induced drag at low speeds, but reduced wing areas would decrease parasite drag at high speeds [57], suggesting that *Alcione* may have been adapted for relatively fast flapping flight compared to other nyctosaurids. Alternatively, reductions in wingspan might represent an adaptation to underwater feeding, i.e., plunge diving of the sort practiced by gannets, tropicbirds, and kingfishers, where smaller wings would reduce drag underwater.

### *Simurghia robusta* gen. et sp. nov.

urn:lsid:zoobank.org:act:CBA04F2E-D7BA-47BC-A76B-1096B1FE4354

**Etymology**. The genus name refers to the Simurgh, a flying beast from Persian mythology. The species name is from the Latin *robusta*, “robust.”

**Fig 10:**
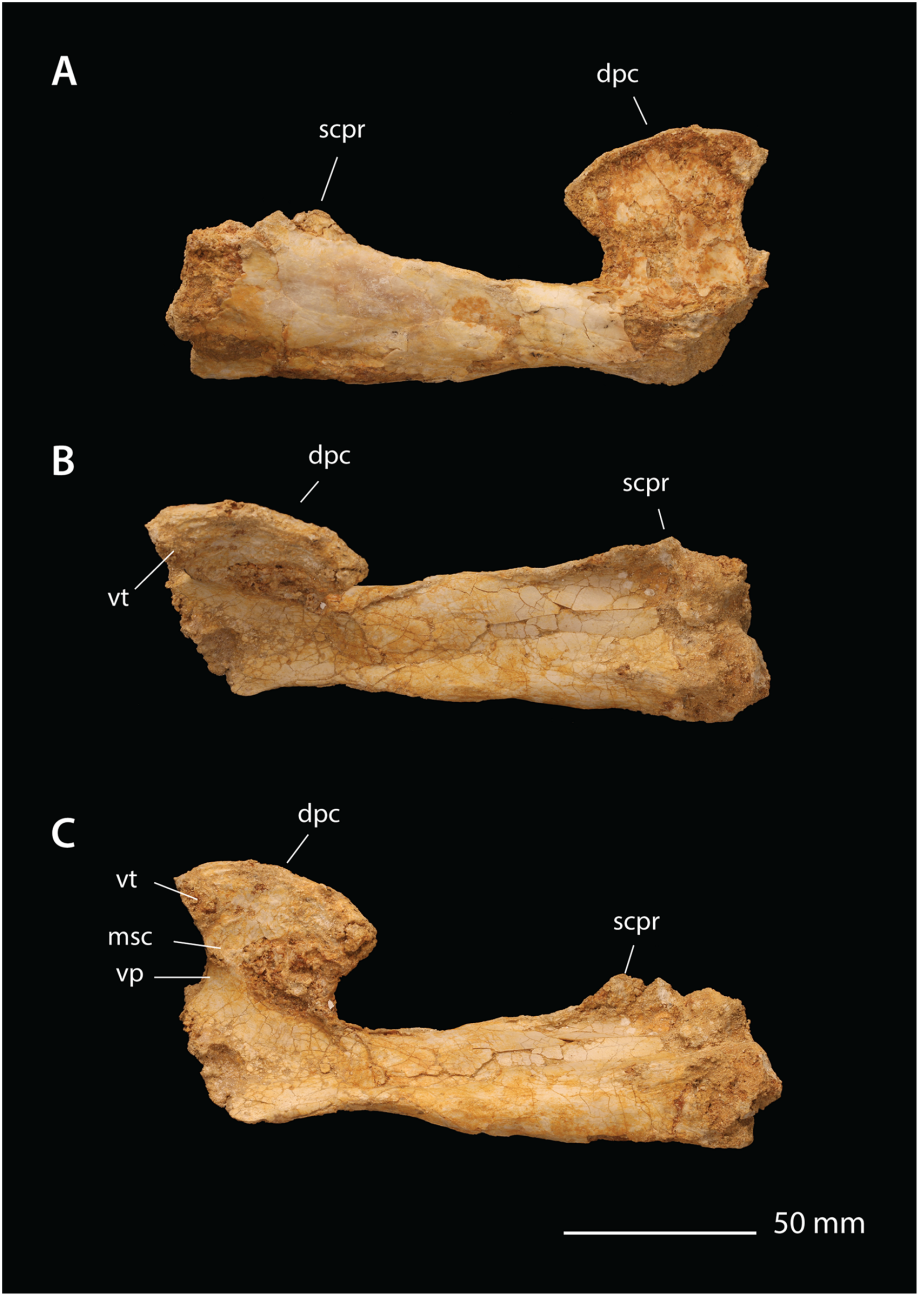
FSAC-OB 7, holotype right humerus, *S*. *robusta*. In (A), dorsal view, (B) ventral view, and (C) posterior view. Abbreviations: dpc, deltopectoral crest; msc, muscle scar; scpr, supracondylar process of the ectepicondyle; vp, ventral pillar; vt, ventral tubercle of the deltopectoral crest.

**Holotype**. FSAC-OB 7 (Fig 10).

**Locality and horizon**. Middle Couche III, Sidi Daoui, Khouribga Province, Morocco.

**Diagnosis**. Large nyctosaurid, with a humerus that is approximately 165 mm long. The deltopectoral crest is proportionately short and broad, but the apex is strongly expanded with a strongly convex apical margin giving it a fan shape. The ventral pillar is shifted to the proximal margin of the deltopectoral crest. The humeral shaft is proportionately robust and distally expanded. The supracondylar process is hypertrophied and triangular.

**Description**. *Simurghia* is a large nyctosaurid most closely resembling “*N*.” *lamegoi* in terms of size and morphology. The humeral head and ulnar crest are broken away. The humeral shaft is robust, with its distal half expanded and subtriangular as in other Pteranodontia. The deltopectoral crest resembles “*N*.” *lamegoi* in being proportionately short and broad, but with a prominent terminal expansion and a strong proximal prong of the apex, such that the proximal margin of the deltopectoral crest is strongly convex. As in other nyctosaurids, there is a strong ridge on the ventral surface of the deltopectoral crest, the ventral pillar, terminating in a tubercle at the apex. However, the ventral pillar lies along the proximal edge of the deltopectoral crest, as in “*N*.” *lamegoi* [2,22], whereas the pillar is more distally located in *Nyctosaurus*, *Alcione*, and *Barbaridactylus*. A muscle scar extends proximodistally across the neck of the deltopectoral crest, again as in *Alcione*.

The distal condyles are not preserved, but there is an unusually large, subtriangular supracondylar process, an autapomorphy of *Simurghia*.

**Discussion**. *Simurghia* is referred to Nyctosauridae on the basis of the deltopectoral crest, which is hatchet shaped, with a ventral pillar, and weakly curved but not warped. It differs from other nyctosaurids in the broad fan-shaped deltopectoral crest (S6 Fig), the position of the ventral pillar along the proximal margin of the crest, and the hypertrophied supracondylar process.

Although *Simurghia* resembles *Alcione*, it is unlikely to represent an adult *Alcione*. Specimens referred to *Alcione* are all subadults or mature: bones have the dense, avascular surface texture that characterizes adult pterosaurs [51], the condyles are well ossified, the holotype scapulocoracoid is fused, and the synsacrum is fused in a referred specimen. *Alcione* humeri average 93 mm (*n* = 12) long and reach a maximum of 102 mm, versus an estimated 165 mm for *Simurghia*. Assuming isometric scaling, *Simurghia* would weigh 560% the mass of the average *Alcione*. Such an extreme size discrepancy exceeds what is expected for intraspecific variation or sexual dimorphism. Furthermore, there are no humeri that are intermediate in length, implying that the sample comes from 2 distinct populations of adults.

Finally, *Simurghia* exhibits features—large size, the very broad, fan-like deltopectoral crest, and the position of the ventral pillar along the medial edge of the deltopectoral crest—that suggest affinities with “*N*.” *lamegoi*, not *Alcione*.

### *Barbaridactylus grandis* gen. et sp. nov.

urn:lsid:zoobank.org:act:626E3A52-74AB-401B-BCD9-8849FD4D43F0

**Etymology**. The genus name refers to North Africa’s Barbary Coast region and the Greek *dactylo*, “finger.” The species name is from the Latin *grandis*, “great.”

**Locality and horizon**. Middle Couche III, Sidi Daoui, Khouribga Province, Morocco.

**Fig 11:**
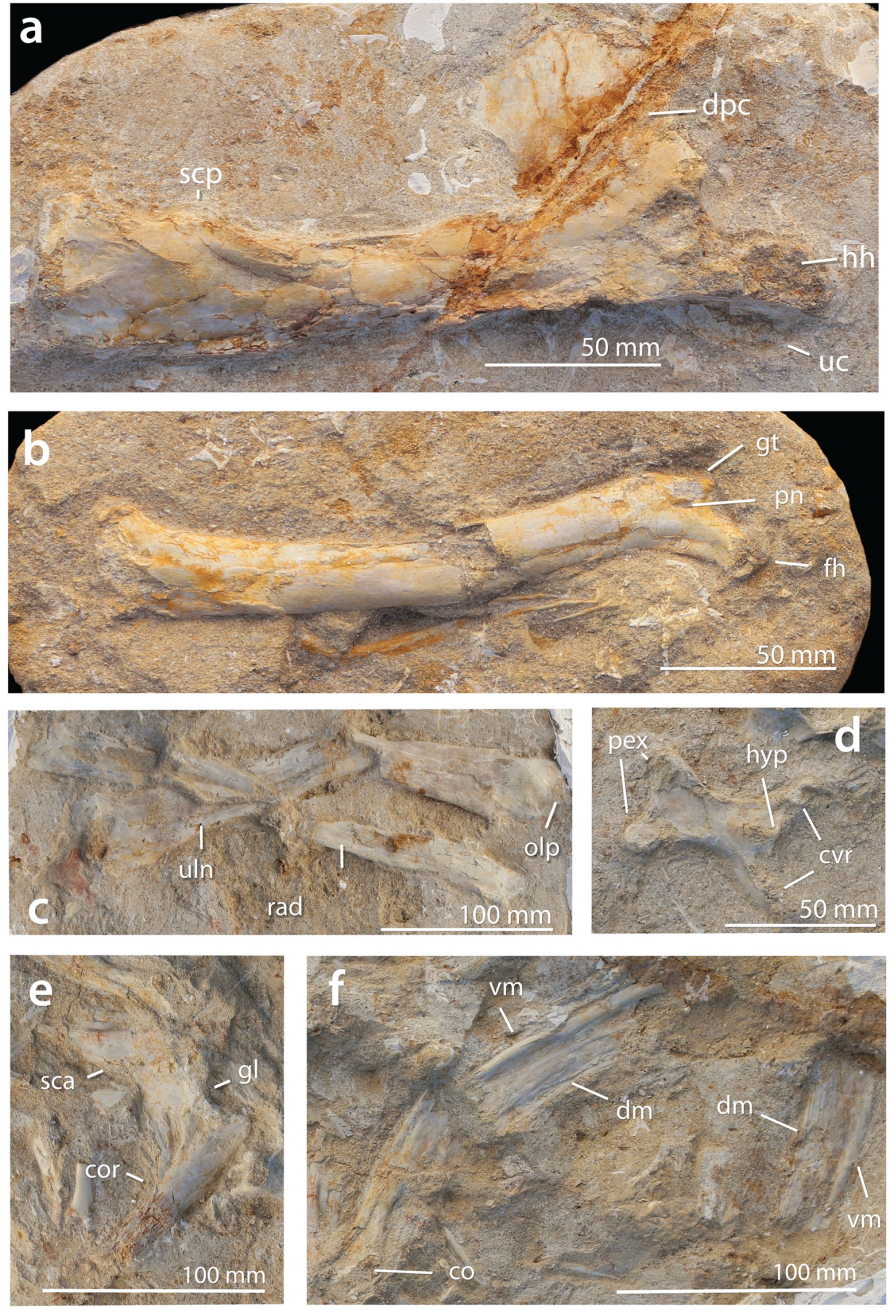
FSAC-OB 232, holotype skeleton of *B*. *grandis*. (A) Left humerus in anterior view, (B) right femur in anterior view, (C) right radius and ulna in posterior view, (D) cervical vertebra in ventral view, (E) left scapulocoracoid in medial view, and (F) posterior ramus of the right mandible in medial view. Abbreviations: co, coracoid; cot, cotyle of mandible; cvr, cervical ribs; dm, dorsal margin of mandible; dpc, deltopectoral crest; gl, glenoid; fh, femoral head; gt, greater trochanter; hh, humeral head; hyp, hypapophysis; olp, olecranon process; pex, postexapophysis; pn, pneumatopore; rad, radius; sca, scapula; scp, supracondylar process of the ectepicondyle; uc, ulnar crest; uln, ulna; vm, ventral margin of mandible.

**Holotype**. FSAC-OB 232 (Fig 11), associated skeleton including left humerus, radius and ulna, right femur, left scapulocoracoid, partial right mandible.

**Fig 12:**
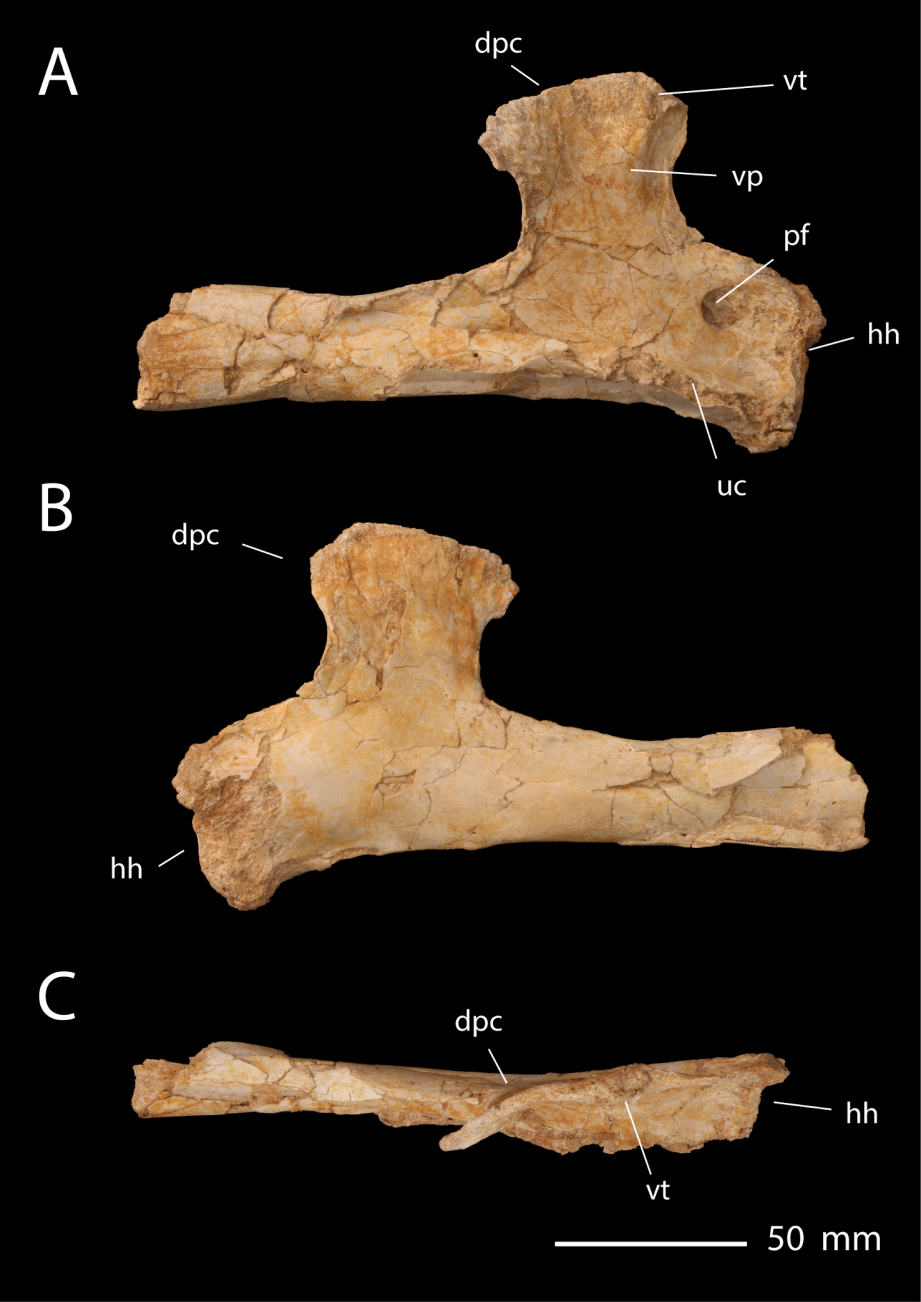
FSAC-OB 8, right humerus of *B*. *grandis*. In (A), ventral view, (B) dorsal view, and (C) anterior view. Abbreviations: dpc, deltopectoral crest; hh, humeral head; uc, ulnar crest; pf, pneumatic foramen; vp, ventral pillar; vt, ventral tubercle.

**Referred specimens**. FSAC-OB 8, 9, 10 (Fig 12), and 11, humeri.

**Diagnosis**. Large nyctosaurid, with a humerus that is up to 225 mm long. The humerus is slender, with the deltopectoral crest well distal to the humeral head. The deltopectoral crest is short, broad, and subrectangular, with weak constriction; warping of the deltopectoral crest is weakly developed. The humeral head has large ventral pneumatic fossa and foramen/foramina. Small pneumatic foramina are proximal to the lateral condyle. The bones of the antebrachium are slender—130% of the humerus’s length. The femur is 85% of the humerus’s length, with a slender shaft and a moderately developed greater trochanter.

**Description**. The type (Fig 11) preserves parts of the left and right mandible. There is a small cotyle posteriorly; ahead of this, the jaw becomes deeper and plate-like, with a thick ventral margin and a sharp occlusal margin. The dorsal margin is gently concave, indicating that the upper and lower jaws were upcurved as in other nyctosaurids.

A single cervical is preserved. It is proportionately short and broad, as in other nyctosaurids [35] and pteranodontids.

The scapulocoracoid is preserved in medial view. It resembles other nyctosaurids, being a boomerang-shaped element with the robust scapula and coracoid meeting at an angle of 60°. The 2 elements are fused, suggesting skeletal maturity [51].

The humeral head has a semicircular dorsal margin and a concave anterior ventral. The humeral shaft (Figs 11A and 12) is long and slender and sigmoidal in anterior view. The deltopectoral crest is distally placed relative to the humeral head, unlike *Alcione* but as in *Nyctosaurus* [35]. The deltopectoral crest is constricted at midlength and distally expanded to give it the characteristic hatchet shape. However, the deltopectoral crest is unusually short and broad. The distally expanded tip, which gives nyctosaurids the distinctive hatchet-shaped crest, is weakly developed, a primitive feature. In anterior view, the crest is slightly warped. This feature is very weakly developed compared to pteranodontoids such as *Pteranodon* but better developed than in other nyctosaurids. Ventrally, there is a prominent ventral pillar running from the apex down the shaft. Anterior to this is a muscle scar, running obliquely to the distal prong of the deltopectoral crest.

The ventral surface of the humeral heads bears a large pneumatic fossa, with either a foramen or several small foramina. The presence, size, and position of this foramen are unique to *Barbaridactylus* among nyctosaurids. The ulnar crest is well developed and subtriangular in shape; it projects ventrally.

Distally, there is an enlarged supracondylar process as in other nyctosaurids. There is a depression between the medial and lateral condyles, with a pneumatic foramen inside the depression, beneath the lateral condyle. A pair of small, elongate pneumatic foramina are present proximal to the lateral condyle, which appear to be unique to *Barbaridactylus* among nyctosaurs.

The antebrachium resembles that of *Nyctosaurus* [35]. The ulna is relatively slender, in contrast to the robust ulna of *Alcione*, and weakly expanded at either end. The radius is about two-thirds of the diameter of the ulna.

The femur resembles other nyctosaurids and pteranodontids [35] in having a sigmoidal shaft with a strong dorsal projection of the humeral head and a weakly developed greater trochanter. In contrast to *Nyctosaurus* [35] and *Alcione*, the femur lacks the strong distal expansion of the shaft. The end of the shaft is more gently expanded, as in *Pteranodon* [35].

**Comments**. *Barbaridactylus* is referred to Nyctosauridae on the basis of the deltopectoral crest, which is hatchet-shaped, with a ventral pillar, and weakly curved rather than warped in end view. It is distinguished from other nyctosaurids by its large size, long and slender humerus, quadrangular deltopectoral crest, and foramen/foramina on the anterior surface of the humerus below the deltopectoral crest. The pneumatic foramen is variable in *Barbaridactylus*, and it differs in size and morphology in all the individuals studied; in some individuals, it is developed as a single foramen, and in others, it is developed as a pair of foramina. Nevertheless, this feature is seen, where exposed, in all specimens referred to *Barbaridactylus* and is absent in other nyctosaur material.

*Barbaridactylus* resembles “*N*.” *lamegoi* from the Campanian-Maastrichtian of Brazil [22] in terms of size and the broad deltopectoral crest and the proximally shifted ventral pillar. These affinities are supported by phylogenetic analysis (see below). However, it lacks the strongly pointed proximal prong of the deltopectoral crest seen in “*N*.” *lamegoi*, indicating that the 2 are distinct.

Azhdarchoidea Kellner 2003 [46]

Neoazhdarchia Kellner 2003 [46]

Azhdarchidae Nessov 1984

### *Phosphatodraco mauritanicus* Pereda-Suberbiola et al. 2003 [43]

**Fig 13:**
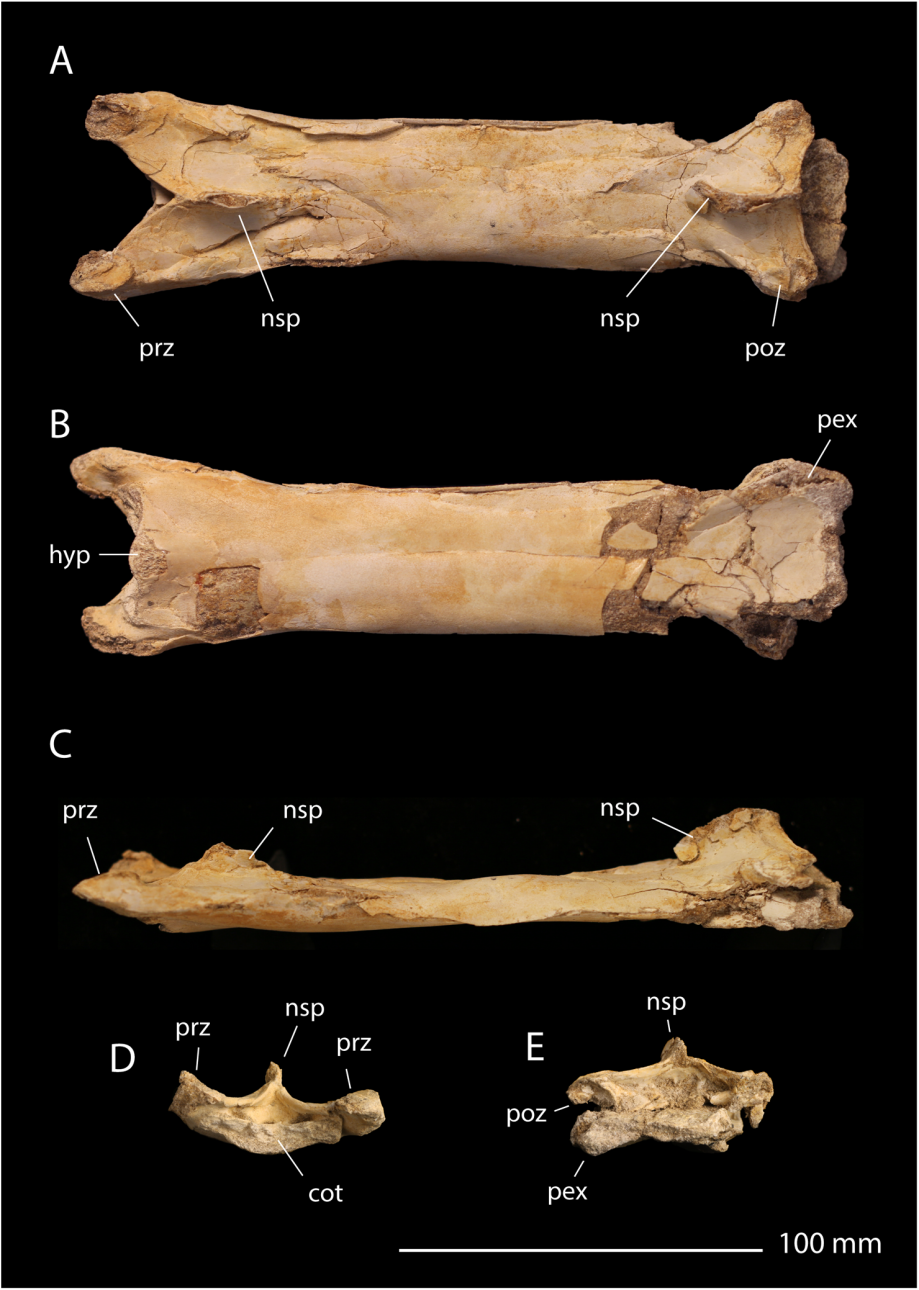
FSAC-OB 12 *P*. *mauritanicus* cervical vertebra. In (A), dorsal view, (B) ventral view, (C) left lateral view; (D) anterior view; (E) posterior view. Abbreviations: cot, cotyle; hyp, hypapophysis; nsp, neural spine; pex, postexapophysis; poz, postzygapophysis; prz, prezygapophysis.

**Referred material**. FSAC-OB 12 (Fig 13), cervical vertebra C5, and FSAC-OB 13, cervical vertebra.

**Description**. The centrum of the referred cervical vertebra (Fig 13) measures 190 mm, and the maximum length of the vertebra is 210 mm. These measurements closely compare with cervical 6 of *P*. *mauritanicus*, which measures 196 mm and 225 mm, respectively [43].

The cervical is long and slender, as typical of Azhdarchidae [21]. The length of the vertebra is approximately 400% the width across the prezygapophyses, matching the proportions of cervical 6 of the holotype of *Phosphatodraco*, OCP DEK/GE 111 [43]. The centrum lacks lateral pneumatic foramina, as in other azhdarchids. Prominent postexapophyses project ventrolaterally.

As in other Azhdarchidae, a hypapophysis projects anteriorly beneath the cotyle [21]. The anterior cotyle is flanked by grooves beneath the prezygapophyses, implying that the cervical ribs had not fused to the centrum to form transverse foramina. The animal may have been near maturity but was not skeletally mature.

The neural arch is reduced and confluent with the body of the centrum. The prezygapophyses project anterodorsally, while the postzygapophyses project almost laterally. The neural spine is reduced as in other azhdarchids [21]; it forms small anterior and posterior blades, but between the 2 blades, the neural spine is not developed.

**Comments**. The azhdharchid *P*. *mauritanicus* has previously been described from the phosphates [43]. The vertebra described here is consistent with *Phosphatodraco* in size and proportions, supporting referral to that taxon.

### Azhdarchidae aff. *Quetzalcoatlus*

**Fig 14:**
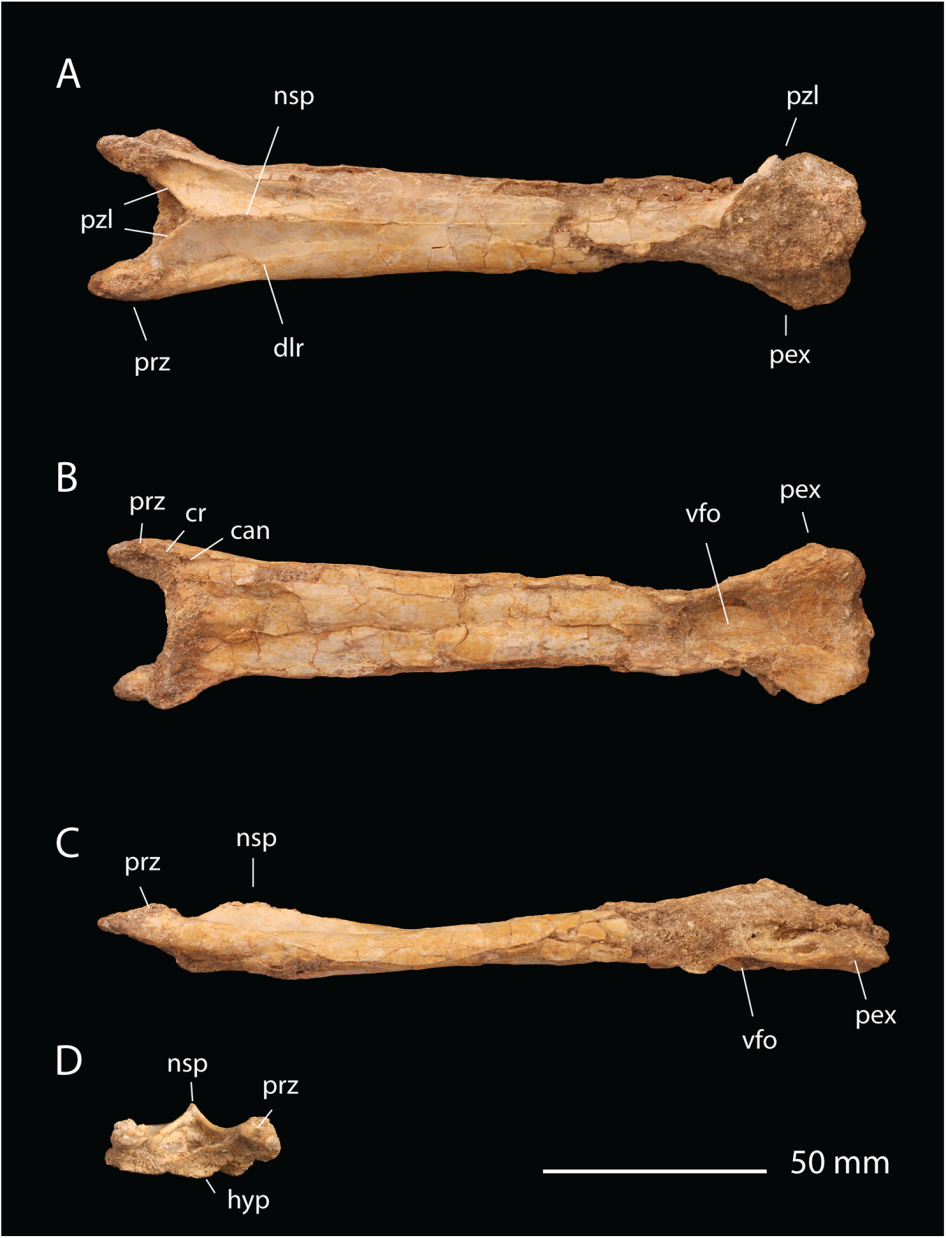
FSAC-OB 14, aff. *Quetzalcoatlus* cervical vertebra. In (A), dorsal view, (B) ventral view; (C), left lateral view, and (D), anterior view. Abbreviations: dlr, dorsolateral ridge; hyp, hypapophysis; nsp, neural spine; pex, postexapophysis; poz, postzygapophysis; prz, prezygapophysis; pzl, prezygapophyseal laminae; vfo, ventral fossa.

**Material**. FSAC-OB 14, cervical vertebra C5 (Fig 14).

**Description**. The cervical vertebra comes from a small azhdarchid; the centrum measures 153 mm. However, the surface bone lacks vascularization, and the cervical ribs are fused to the centrum. These features indicate that despite the animal’s small size, it was somatically mature [51].

The centrum is typical of Azhdarchidae in being elongate and lacking pneumatic foramina piercing the lateral surfaces. The elongate proportions of the vertebra indicate that it comes from the middle of the neck. Based on comparisons with *Quetzalcoatlus* [58], the vertebra is probably C4 or C5, most likely C5 given the similarities in shape. Centrum length is 440% of the width across the prezygapophyses, more elongate than the C5 of *Phosphatodraco* [43], where length is 356% of the prezygapophyseal width.

The centrum differs from *Phosphatodraco* in being broad anteriorly but very narrow posteriorly; however, this feature is typical of *Quetzalcoatlus* [59]. Posteriorly, the centrum bears a deep ventral depression; a similar depression is present but much more weakly developed in *Phosphatodraco*. Lateral to the depression, a pair of prominent, subtriangular postexapophyses project laterally. Those of *Phosphatodraco* are more weakly developed.

As in other Azhdarchidae, the neural arch is confluent with the centrum, with no clear separation between them. Anteriorly, the prezygapophyses are long and narrow, while those of *Phosphatodraco* are shorter and more ovoid. Two laminae extend from the prezygapophyses to the neural spine, forming a “V.” A similar feature is seen in *Phosphatodraco*, but here the ‘V’ extends further back so that the dorsal surface of the centrum is broadly exposed, which does not occur in *Phosphatodraco*. A pair of faint ridges extend back from the prezygapophyses onto the dorsal surface of the vertebra. Postzygapophyses are laterally projecting and shifted anteriorly.

The neural spine is highly reduced as in other azhdarchids. Anteriorly, it forms a low ridge, which then becomes shallower posteriorly until near the middle of the centrum it forms a faint line running along the dorsal surface of the centrum.

**Comments**. The new azhdarchid is smaller than *Phosphatodraco* but exhibits fusion between the cervical ribs and centrum and an avascular surface bone texture. This suggests that it is mature [51] and not a juvenile *Phosphatodraco*. The new azhdarchid also differs from *P*. *mauritanicus* in the strong tapering of the centrum posteriorly, the deep ventral fossa on the posterior end of the centrum, and the larger postexapophyses. These are derived features that are shared with *Quetzalcoatlus* [59] to the exclusion of *Phosphatodraco* and suggest affinities with that genus. Phylogenetic analysis places this azhdarchid as sister to a clade including *Zhejiangopterus linhaiensis*, *Arambourgiania philadelphiae*, *Hatzegopteryx thambena*, and *Quetzalcoatlus* spp.

### Sidi Chennane Azhdarchid

**Fig 15:**
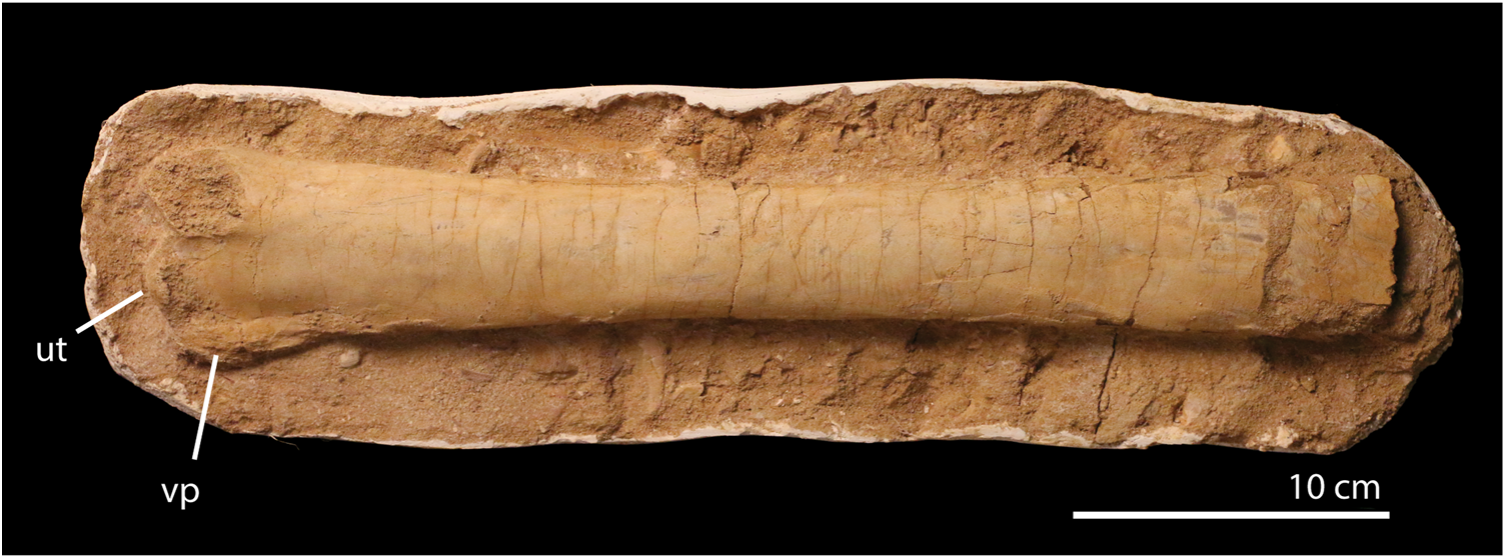
FSAC-OB 203, giant azhdarchid (?*Arambourgiania*) ulna. Middle shaft and distal end of the left ulna, in posterior view. Abbreviations: ut, ulnar tubercle; vp, ventral process.

**Material**. FSAC-OB 203, left ulna missing proximal end (Fig 15).

**Description**. The ulna comes from a very large pterosaur. The preserved part of the bone, comprising the distal end and the middle of the shaft, measures 362 mm in length and may have been 600–700 mm long when complete. The shaft is 40 mm in diameter at its narrowest and 65 mm at its distal end. These proportions suggest a wingspan approaching 9 m.

Overall, the shaft is proportionately long and slender. The shaft is broad proximally, narrows distally, and then gradually expands towards its distal end. The distal end bears a broad tubercle, as in *Azhdarcho* [21]. The ventral margin bears a long, low flange, again as in *Azhdarcho*. There is an articular surface dorsal to the tubercle, separated by a notch.

**Comments**. The broad tubercle and low, proximodistally elongate ventral crest support azhdarchid affinities; the tubercle of Pteranodontidae is smaller, the ventral crest is much more pronounced, and the ulna is proportionately shorter and broader.

Affinities with either *Phosphatodraco* or aff. *Quetzalcoatlus* appear unlikely given that the bone texture of both indicates that they are somatically mature. Affinities with or referral to the giant azhdarchid *A*. *philadelphiae* seem more likely given that both are known from late Maastrichtian deposits of the Tethys sea [37], but more complete material is needed to test this assignment. The animal approached *Quetzalcoatlus* in size, but it was much more lightly built and presumably weighed much less. These proportions presumably indicate a distinct flight mode and ecological niche, suggesting that giant pterosaurs occupied a range of niches.

### Phylogenetic analysis

**Fig 16:**
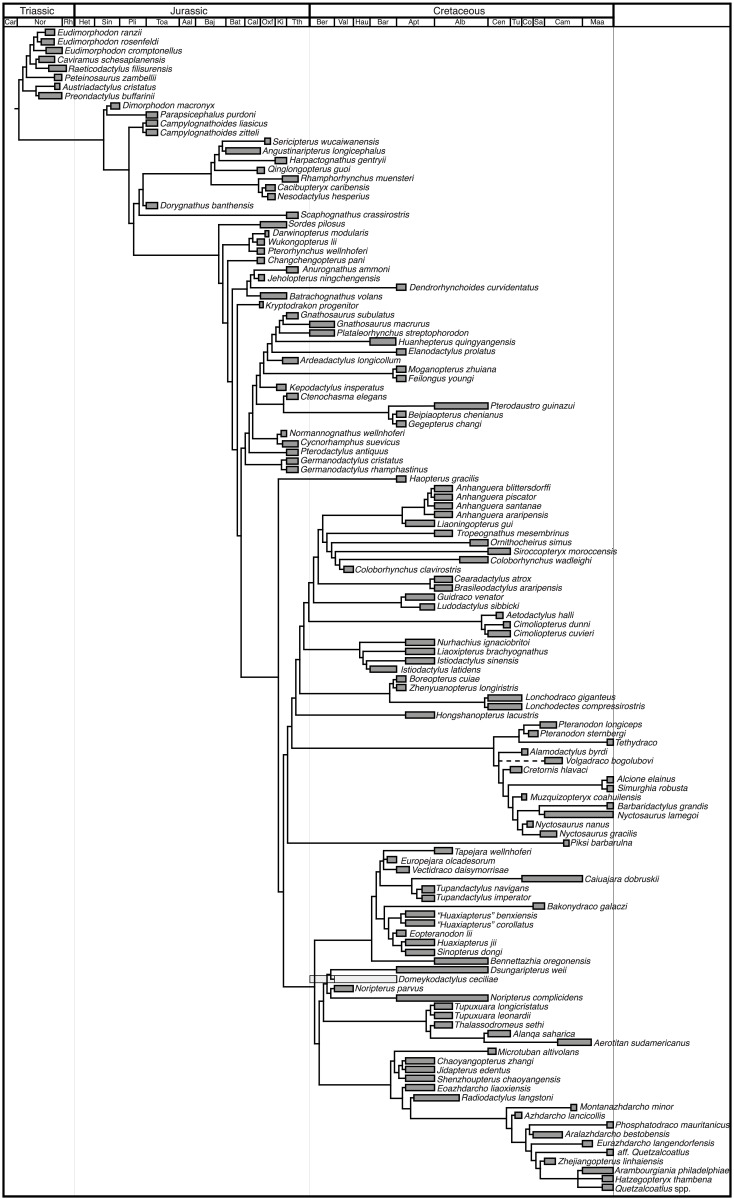
Time-calibrated phylogenetic analysis. Tree showing placement of *T*. *regalis*, *A*. *elainus*, *S*. *robusta*, *B*. *grandis*, *P*. *mauritanicus*, and aff. *Quetzalcoatlus*. Maximum parsimony analysis of the character-taxon matrix (S1 Data) recovered 4 most parsimonious trees with length 1,126.651 (consistency index = 0.336, retention index = 0.793). Divergence dates are set at 1 Ma, and ranges show the resolution of stratigraphic dating.

**Fig 17:**
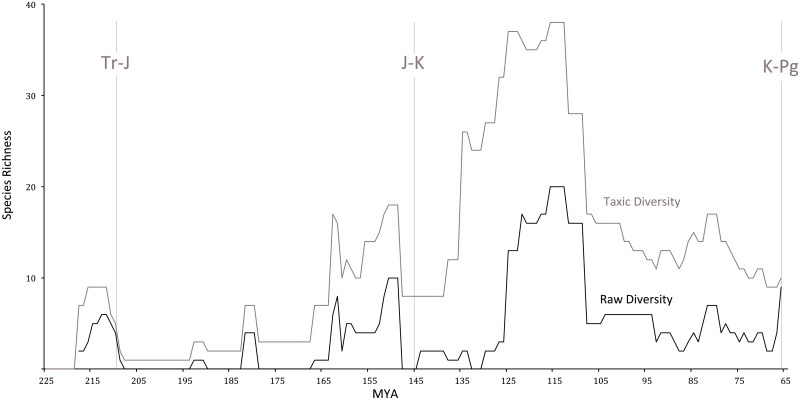
Diversity curve showing taxic (raw) diversity and phylogenetic (with ghost lineages) diversity of pterosaurs over time (millions of years ago [MYA]). Late Cretaceous taxic diversity remains relatively steady up to the Cretaceous–Paleogene (K-Pg) boundary following the mid-Cretaceous diversity drop. The slight decrease in phylogenetic diversity in the Late Cretaceous is the expected result of the Signor-Lipps Effect: ghost taxa cannot be inferred for the Late Maastrichtian because no post-Cretaceous pterosaurs exist to create ghost lineages; here phylogenetic diversity equals taxic diversity. Abbreviations: Tr-J, Triassic-Jurassic; J-K, Jurassic-Cretaceous.

We undertook a morphological phylogenetic analysis of the new Moroccan species and all diagnostic Late Cretaceous species based on a previous character-taxon matrix [60] (Fig 16). Curves of taxic diversity (raw species counts over time) and phylogenetic diversity (species plus ghost lineages) were generated from the results, with minimum divergence times used (Fig 17).

*Tethydraco* is recovered as a pteranodontid while *Alcione*, *Simurghia*, and *Barbaridactylus* are recovered as nyctosaurids. *Phosphatodraco* and the new small azhdarchid are recovered as different lineages within Azhdarchidae.

In the current analysis, 3 or perhaps 4 other lineages extend into the final 25 million years of the Cretaceous. These include 2 lineages of Tapejaromorpha, 1 represented by *Caiuajara* and 1 represented by *Bakonydraco*, and a third lineage giving rise to *Piksi*. The Cenomanian *Alanqa saharica* and the Campanian-Maastrichtian *Aerotitan sudamerica* are recovered as thalassodromids, which would imply survival of thalassodromids into the latest Cretaceous. Both are known from very fragmentary material, and thus, these results are not well supported and will require testing by the recovery of more complete remains.

### Functional diversity

To test the hypothesis of a Late Cretaceous decline in pterosaur diversity, we assessed pterosaur functional diversity in the final 20 Ma of the Cretaceous. In contrast to disparity, which measures the range of morphologies regardless of function, functional diversity uses functional characters or correlates to quantify the diversity of function [61,62], e.g., inferred diet, locomotor style, or habitat. Functional correlates used here include morphological features such as size, jaw shape, or limb proportions that are likely to correlate with or influence function. Based on analogy with living birds, jaw shape likely reflects diet, wing proportions will reflect locomotion, and body size is likely to be associated with a wide range of variables including diet, locomotion, physiology, and life history. Given the incompleteness of the fossils and the difficulty of inferring function, functional diversity cannot capture the full range of ecological niches but can act as a proxy for niche occupation.

Functional diversity has advantages over taxon counting. First, it should provide a more accurate picture of the range of ecological niches and ecosystem structure than simply counting species [63], because species richness need not correlate with niche occupation. Second, because functional diversity measures dissimilarity among fossils, rather than using the number of taxonomic divisions as a proxy for diversity, it is less affected by taxonomic lumping or splitting. Furthermore, because many species can occupy the same niche, one need not find all or even most taxa to provide a relatively complete picture of ecosystem structure; one need only find representatives of each niche. It follows that functional diversity should be robust to sampling versus taxon counting, which is particularly important with poorly studied groups such as pterosaurs.

To quantify functional diversity, we created a matrix combining discrete and continuous characters to capture ecological and functionally significant morphological variation. Characters include habitat (continental, brackish, or marine), size (wingspan), and morphological characters including jaw curvature, cervical elongation, hindlimb elongation, ulna:humerus ratio, and metacarpal IV:humerus ratio. Wingspan and limb ratios were treated as continuous characters, whereas others were treated as discrete. Functional morphospaces can effectively characterize functional diversity with as few as 4 characters [61]; thus, the use of relatively few characters is appropriate here. Data were taken from fossils described in this study, previous studies of pterosaur size [18], and the literature (S2 Data). Some characters are conserved within clades, e.g., azhdarchids all have elongate cervicals, and thus, based on phylogenetic inference, it is possible to code them when the character is not preserved.

Functional diversity was quantified using principal coordinates analysis (PCoA). PCoA is used instead of principal component analysis (PCA) because unlike PCA, it can use both discrete and continuous characters, and it can accommodate missing data. PCoA rotates and replots data points along a series of axes that summarize the dissimilarity of the dataset. Gower’s distance was used to calculate the distance between points. Calculations were done in PAST [64].

Pterosaurs were assigned to 2 time bins: Santonian-Campanian and Maastrichtian. This was done because the Campanian has relatively few marine pterosaurs, which might artificially depress diversity. The 2 bins span unequal intervals (6.1 Ma versus 14.2 Ma) [65]. Because the Santonian-Campanian interval encompasses over twice the amount of time as the Maastrichtian, time averaging across this interval should artificially increase diversity and should therefore bias the result in favor of higher pre-Maastrichtian diversity.

**Fig 18:**
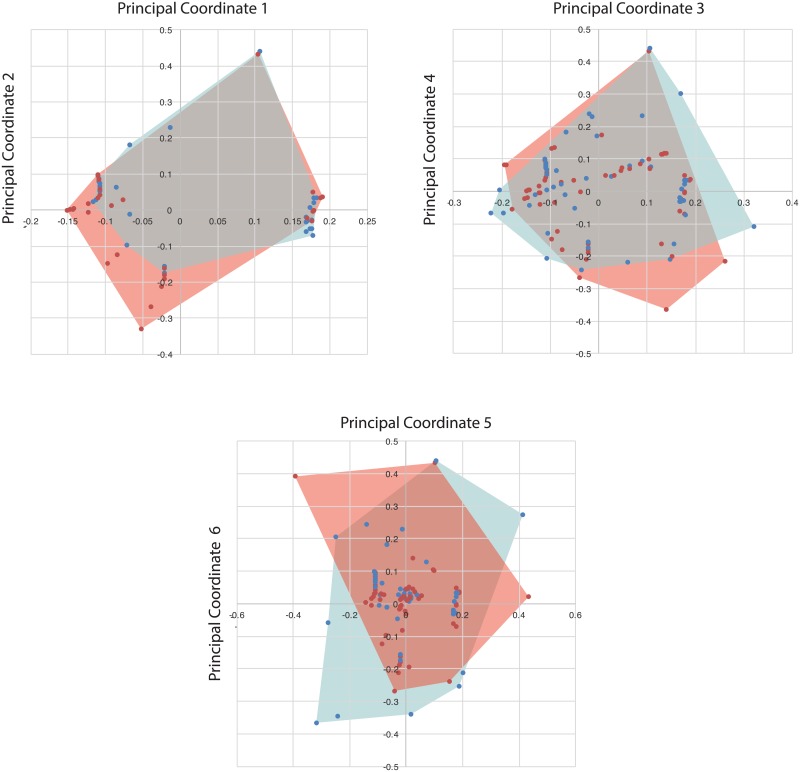
Late Cretaceous pterosaur functional diversity. Santonian-Campanian (blue) versus Maastrichtian (red) functional diversity, based on principal coordinates analysis of habitat, wingspan, jaw shape, and limb proportions (S2 Data). Axis 1 accounts for 54.4% of variation; axis 2, 11.2%; axis 3, 4.5%; axis 4, 3.0%, axis 5, 1.9%; axis 6, 1.4%; and axis 7, 1%.

Our results suggest that despite this, Maastrichtian niche occupation is comparable to or higher than that of the Campanian-Santonian interval (Fig 18). This is true whether functional diversity is quantified in terms of the product of ranges (Maastrichtian = 0.01545, Campanian = 0.01141), proportional to the volume of functional space occupied, or sum of ranges (Maastrichtian = 4.0078; Campanian = 3.7993), proportional to the total spread along the various functional axes. Most of the Santonian-Campanian functional space is also occupied by the Maastrichtian taxa, but new functional space is occupied in the Maastrichtian by giant Azhdarchidae such as *Quetzalcoatlus* and *Hatzegopteryx* in continental ecosystems and *Arambourgiania* and the Sidi Chennane giant in marine environments, driving an increase in functional occupation.

**Fig 19:**
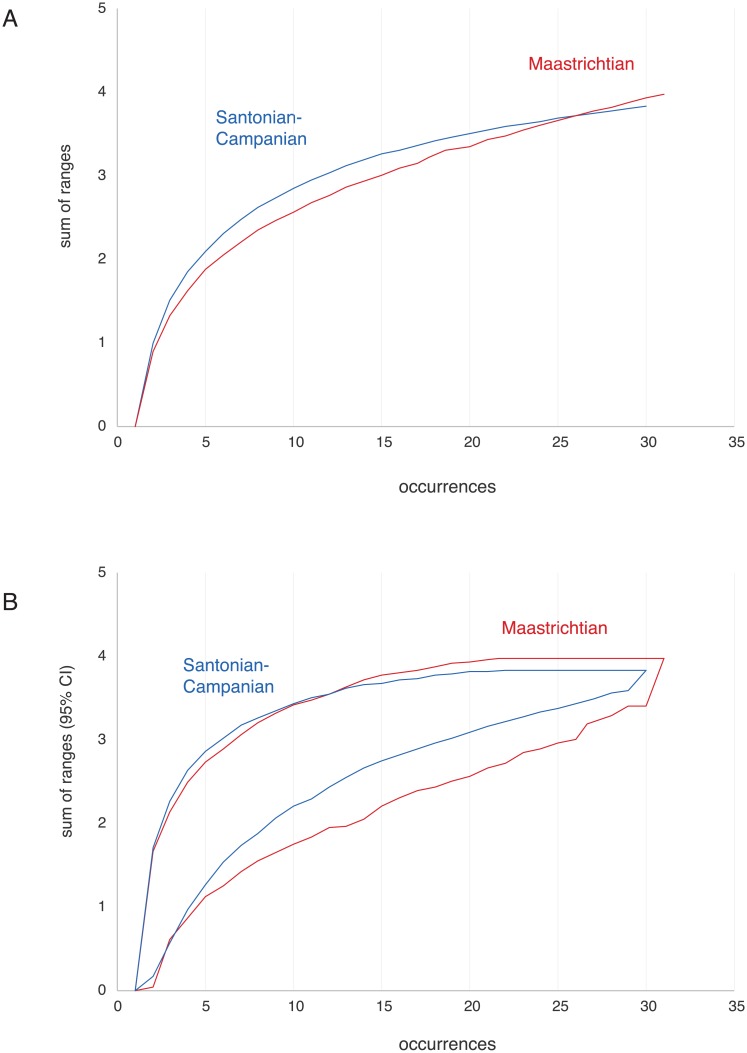
Resampling of functional diversity. Rarefaction of sum of ranges for the Maastrichtian (red) and Santonian-Campanian (blue), showing mean functional diversity (A) and 95% confidence intervals (B).

To test whether the higher diversity of the Maastrichtian is driven by sampling, we rarefied the data, randomly resampling without replacement from the set of occurrences 5,000 times at varying levels of sampling using a custom script in R and then calculating the average functional diversity and 95% confidence intervals at various levels of sampling (Fig 19). Both Maastrichtian and Santonian-Campanian functional diversity increase rapidly with sampling and fail to asymptote. This, perhaps unsurprisingly, implies that Late Cretaceous pterosaur diversity remains undersampled and that further sampling is likely to reveal that pterosaurs occupied a wider range of niches than currently known. Sampling effects are extreme in both intervals given the high diversity of pterosaurs and poor sampling (Fig 19B); the 95% confidence intervals estimated strongly overlap. Our analyses therefore show that there is no support for a decline in pterosaur diversity from the Santonian-Campanian into the Maastrichtian. However, given the strong sampling effects seen, the increased diversity of the Maastrichtian could represent a sampling effect. Further data are needed to test the hypothesis of a Maastrichtian diversity increase. However, the Maastrichtian appears to be more poorly sampled than the Santonian-Campanian as shown by the steeper slope of the sampling curve towards the right end of the graph. If so, increased sampling may increase rather than decrease the difference between the 2 intervals.

## Discussion

### Maastrichtian pterosaur diversity

The new pterosaurs from Morocco, with previously described pterosaurs, indicate that diversity was high and niche occupation may have increased in the late Maastrichtian. Pterosaurs were a diverse and important part of Cretaceous ecosystems up to the K-Pg boundary, consistent with a catastrophic extinction driven by the Chicxulub impact. Three families, Pteranodontidae, Nyctosauridae, and Azhdarchidae, are now known from the late Maastrichtian (Fig 16). Nyctosauridae not only survived into the late Maastrichtian but did so at high diversity. Other evidence for Maastrichtian nyctosaur diversity comes from an isolated femur from the early-mid(?) Maastrichtian Peedee Formation in Maryland [66]. It is referred to Nyctosauridae on the basis of its small size, massive femoral neck, and distally expanded femoral shaft. The Peedee nyctosaurid differs from *Nyctosaurus* in having a robust shaft, but this feature is shared with *Alcione* (Fig 7), suggesting affinities with that genus.

Azhdarchidae are the most diverse group, perhaps because they occur in terrestrial and marine strata, allowing them to occupy more niches and increasing preservation potential. As many as 10 late Maastrichtian species are known. These include *Quetzalcoatlus northropi* [59] *Q*. sp. [59] and an unnamed species [2] from the Javelina Formation of Texas, a slender-necked azhdarchid from the Hell Creek Formation of Montana [14], a small azhdarchid from the Lance Formation of Wyoming [67], *A*. *philadelphiae* from Jordan [37], *H*. *thambema* [68,69] from Romania, a giant species from Mérignon, France [70], and *P*. *mauritanicus* and aff. *Quetzalcoatlus* from the marine phosphates of Morocco [43]. The giant Sidi Chennane azhdarchid could represent *Arambourgiania* or another species.

**Fig 20:**
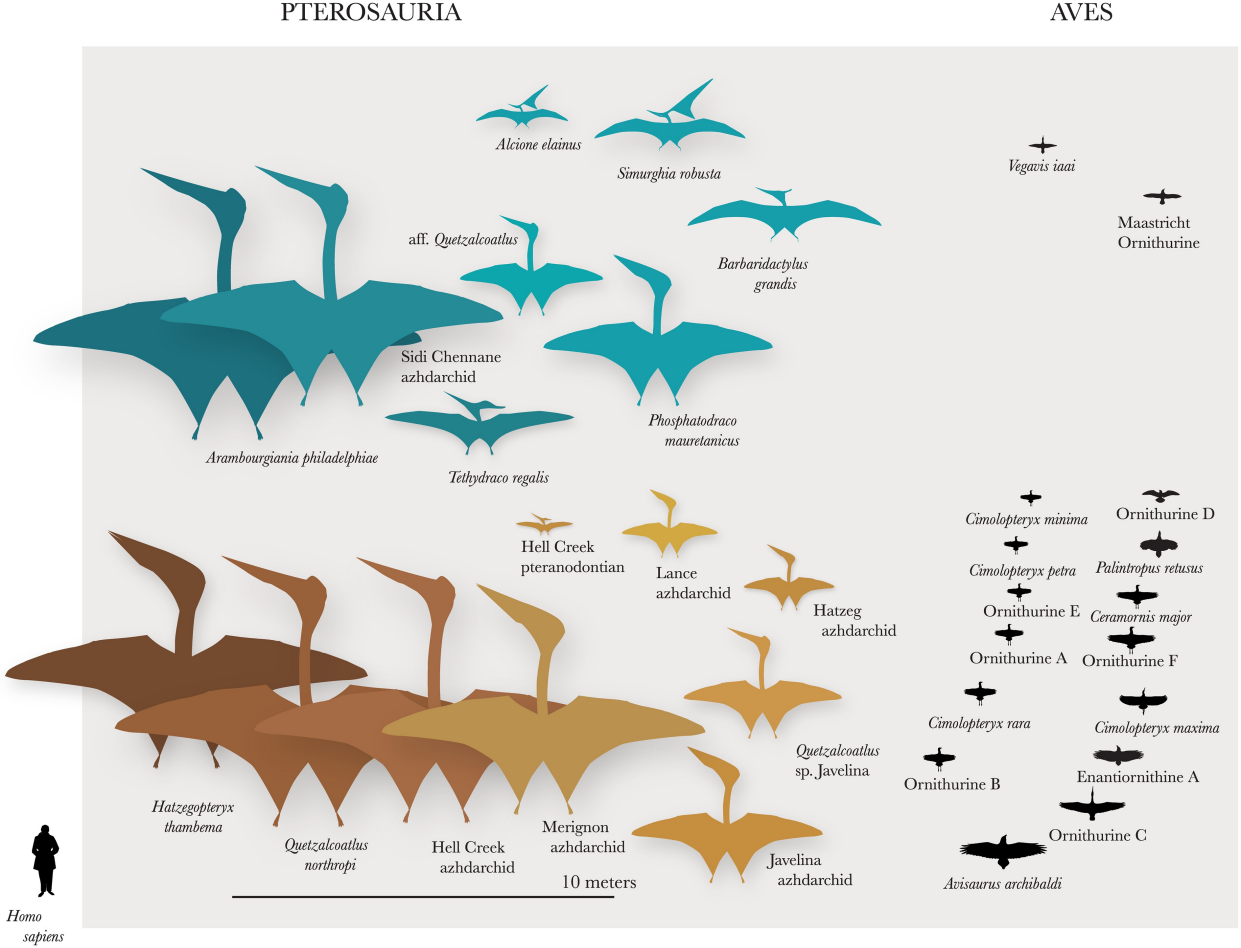
Size disparity of late Maastrichtian pterosaurs and birds. Maastrichtian pterosaurs are larger than coeval birds in both marine (blue) and terrestrial/freshwater (orange) ecosystems. Wingspan estimates for pterosaurs are from S2 Data. Wingspans for terrestrial birds were made using estimated masses from Longrich et al. [74] and the equation for passeriformes from Norberg [75] or from reconstructions based on fossils [76,77].

Maastrichtian pterosaurs both occupied a range of habitats and show a range of morphologies, suggesting diverse ecologies (Figs 18 and 20). Among pteranodontidians, a diversity of wing morphologies implies a wide range of flight styles. Pteranodontids and nyctosaurids were medium-to-giant marine forms with high-aspect ratio wings and low wing loading [71]. Nyctosaurids resemble frigatebirds, marine thermal soarers, in morphology and flight performance, and probably exploited flap gliding and thermal soaring [71]. However, the short-winged *Alcione* may have been better adapted for flapping flight, whereas the large, slender-winged *Barbaridactylus* was probably specialized for gliding. *Pteranodon* was most likely a glider specialized to exploit marine thermals [71,72], and *Tethydraco* probably had a similar ecology. Like modern seabirds, including frigatebirds, gulls, petrels, and tropicbirds, pteranodontids and nyctosaurids would have flown long distances in search of food, feeding on the wing [72] and perhaps while floating on the water [73].

Azhdarchids had short wings but long arms and legs and likely foraged on the ground or wading in shallow water, as suggested by trackways [58,78]. Their ecology is controversial, and azhdarchids have variously been interpreted as scavengers, piscivores, probers, skimmers, or terrestrial predators [58]. Repeated occurrences of azhdarchids in brackish water and marine environments [36,37,43] suggests at least some species exploited marine resources. The short wings of azhdarchids [58] would have been inefficient for aerial foraging; instead, the long, pointed jaws [59] and long necks of azhdarchids, especially *Quetzalcoatlus* and kin, are consistent with hunting while wading in shallow water [79,]. Azdharchids might have fished by grasping prey with the beak, as in storks, or using heron-like spear fishing [73]. Probe traces associated with azhdarchid tracks [80] suggest a stork-like foraging strategy for at least some species. Azhdarchidae are also well represented in continental deposits, however, [81], and some species had short necks [69,78] and robust beaks [2], suggesting a distinct feeding strategy. Similarly, azhdarchid skeletons imply variation in their flight. The sheer size of the largest azhdarchids implies a flight style distinct from that of the smaller species, but variation is seen even within giant azhdarchids, from slender wing bones in the Sidi Chennane giant, to light but robust bones in *Quetzalcoatlus* [49], to the massively built *Hatzegopteryx* [78], showing that diverse locomotor strategies existed even within the giants. Given variation in beak shape [78], neck proportions [78], wing proportions, and body size, as well as their occurrence in a range of environments, azhdarchids probably exploited a variety of niches; they are best seen not as a specialist lineage but as a Late Cretaceous adaptive radiation.

The closest analogues to azhdarchids among modern organisms are long-necked, long-beaked birds such as cranes, herons, and bustards. Rather than being highly specialized for a particular niche, these birds are opportunistic feeders and occupy a wide range of niches [56,82–84]. By analogy, it is unlikely that azhdarchids specialized on a single niche. Instead, some may have foraged for vertebrates and invertebrates in marginal marine habitats such as bays, lagoons, mudflats and estuaries, like many herons and storks [83]. Others may have been terrestrial predators [69,78], similar to the cattle egret, white stork [83], and ground hornbill [84]; scavengers [69] analogous to the Marabou stork; or plant-dominated omnivores similar to bustards and cranes [82].

These qualitative assessments are borne out by quantitative analyses of pterosaur functional diversity (Figs 18 and 19). PCoA of functional characters suggests that Maastrichtian pterosaurs occupied a range of ecologies, and resampling suggests that functional diversity is undersampled (Fig 19). Just as sampling the Moroccan phosphates has revealed previously unknown diversity, future finds in other localities may reveal previously unrecognized taxic and ecological diversity.

### Diversity dynamics preceding the K-Pg boundary

Our data, along with previous discoveries, show that pterosaur diversity did not decline in the latest Cretaceous and may have been increasing prior to the K-Pg extinction (Figs 16–19). Taxic diversity peaks in the Early Cretaceous with a pterodactyloid radiation, followed by a decline in the middle Cretaceous. Subsequently, however, pterosaurs radiated up to the K-Pg boundary; Azhdarchidae diversified in terrestrial and nearshore marine environments, while Pteranodontia radiated in nearshore and offshore marine habitats. Similarly, pterosaur functional diversity appears to actually increase in the latest Cretaceous. This increase is driven primarily by the appearance of giant azhdarchids such as *Quetzalcoatlus* and *Arambourgiania*, but the appearance of the short-winged *Alcione* and the large *Barbaridactylus* shows nyctosaurs radiated as well.

The apparent latest Cretaceous decline of diversity [8] and disparity [9] seen in previous studies results from the Signor-Lipps Effect and variation in the completeness of the fossil record. Similarly, while phylogenetic diversity estimates show a modest decline (Fig 17), this is a predictable result of the Signor-Lipps Effect: the absence of post-Cretaceous pterosaurs means that ghost lineages cannot be inferred in the late Maastrichtian. Again, the patterns recovered here support a catastrophic extinction at the K-Pg boundary.

Given the rarity of pterosaur fossils, it is possible that additional lineages survived up to the K-Pg boundary. Several candidates exist. One such clade is Tapejaridae. Previously restricted to the Early Cretaceous, *C*. *dobruskii* [16] from the Turonian-Campanian of Brazil shows that tapejarids survived into the Late Cretaceous. Our analysis also follows previous analyses [24,85] in recovering *B*. *galaczi*, previously referred to Azhdarchidae [23], as a Late Cretaceous tapejarid.

Another candidate lineage is represented by *P*. *barbarulna* from the Campanian of Montana [25]. *Piksi* is controversial; first described as a bird, it has been reinterpreted as a small pterosaur [20] and then as a theropod [86]. *Piksi* shows no characters of birds or theropods that are not also seen in pterosaurs [20] but does show pterosaur synapomorphies and autapomorphies (see S1 Text). *Piksi* may represent a distinct lineage of small pterosaur that extends into the late Cretaceous. If so, it is unclear whether it belongs to a known family or a previously unknown lineage.

A third clade that may extend into the latest Cretaceous is Thalassodromidae. Our analysis recovers the Cenomanian *A*. *saharica* [87] and the Campanian/Maastrichtian *A*. *sudamericanus* [88] as thalassodromids. This result is poorly supported, and given the lack of other latest Cretaceous thalassodromiids, these fossils could represent azhdarchids.

Extending these clades to the K-Pg boundary would involve range extensions of 10–30 Ma. However, as shown by the discovery of late Maastrichtian Pteranodontidae, the Signor-Lipps Effect exerts a powerful influence on taxa with a poor fossil record. Long range extensions are possible with poor sampling, and the terrestrial record is particularly incomplete. Here most pterosaurs are known from rare, dissociated remains [14,67,69,88,89], and few associated specimens are known [25,59,90]. Given this, we hypothesize that pterosaur diversity remains undersampled and predict that further sampling will reveal additional lineages in the late Maastrichtian.

### Avian radiation and pterosaur extinction

Birds did not drive pterosaurs extinct directly. Rather than competing, pterosaurs and birds appear to engage in size-based niche partitioning, avoiding competition. No known Late Cretaceous birds exceeded 2 m in wingspan or a few kg in mass [18,91]. Meanwhile, Late Cretaceous pterosaurs were mostly large bodied, ranging from 2 to over 10 m in wingspan [18], with the possible exception of *Piksi*. This pattern holds in marine ecosystems, where small Ichthyornithes coexisted with large pteranodontids and nyctosaurids, and terrestrial habitats, where small birds [18,91] lived alongside large and giant azhdarchids (Fig 20). Birds apparently outcompeted pterosaurs at small sizes, but the absence of large (>5 kg) birds suggests that the birds could not compete with pterosaurs at large size; here, pterosaurs dominated. This is not to say that no large birds or small pterosaurs existed, but they must have been rare to escape discovery.

A similar pattern is seen with nonavian dinosaurs and mammals. Dinosaurs occupied large-bodied niches as predators and herbivores, and mammals diversified at small body sizes [92]. The fate of the pterosaurs also mirrors the fate of the dinosaurs. Birds and mammals, which were most diverse at small sizes, survived the K-Pg extinctions; pterosaurs and nonavian dinosaurs, which were most diverse at large sizes, did not. While avian competition may not have directly driven pterosaurs extinct, the absence of small pterosaurs resulting from avian competition may have left pterosaurs vulnerable to an extinction event that selected against large size [18].

Finally, pterosaur extinction may have contributed to avian radiation. The extinction of pterosaurs and archaic birds, including enantiornithes and stem ornithurines [74,93], left the surviving birds with few competitors. This created an opportunity for the emergence of a diverse fauna of birds in the early Paleogene [94–96]. Strikingly, within 10 million years of the extinction of the pterosaurs, marine birds diversified. Tropicbirds [97] and the first large marine soaring birds, the Pelagornithidae, appeared in marine ecosystems [98,99], large soaring pelecaniforms appeared in freshwater habitats [100], and large lithornithid palaeognaths appeared in terrestrial habitats [101]. These patterns suggest that the extinction of pterosaurs in these environments allowed birds to evolve large size.

### Conclusions

The high diversity seen in the late Maastrichtian of Morocco suggests that pterosaur diversity remained stable or increased prior to the end-Cretaceous mass extinction. These patterns are consistent with a catastrophic extinction of pterosaurs at the K-Pg boundary, driven by the Chicxulub impact. Pterosaurs and birds engaged in size-based niche partitioning, and pterosaur extinction provided a competitive release that helped drive avian radiation in the Early Cenozoic.

## Materials and methods

### Phylogenetic analysis

Phylogenetic analysis used the character-taxon matrix from Andres et al. [60], updated with new characters and all diagnostic taxa from the Late Cretaceous, for a total of 134 taxa and 271 characters. Ordered and unordered characters were used and equally weighted. Continuous characters were rescaled to unity using the “nstates stand” command. Inapplicable features were reductively coded [102], with multistate coding used to denote variation within species or instances in which all but a couple of the possible states could be excluded. Analysis was conducted with TNT v.1.5 [103] using discrete and continuous character partitions. Ambiguous branch support was not used, zero-length branches were automatically collapsed, and the resultant trees were filtered for best score. Basic tree searches of 2,000 random addition sequence replicates were conducted with and without the parsimony ratchet. For time calibration, the timescale is from Gradstein et al. [104], and ages for species were taken from the literature.

### Diversity curves

Diversity curves were created from the time-calibrated phylogeny. The timescale was converted into 1 Ma bins and divided into stages and early, middle, and late substages. A taxic diversity curve of species counts and a phylogenetic diversity curve including species and ghost lineage counts were generated. A species was counted as present for the entire length of its possible occurrence, with the exception of *Domeykodactylus ceciliae* [105], which is only constrained to the Early Cretaceous and was given the earliest occurrence of its relative *Noripterus parvus* [106]. Minimum divergence dates of 0 million years were used for the ghost taxon and lineage extensions.

## Supporting information

S1 Data Character-taxon matrix for phylogenetic analysis. (TXT)

S2 Data Functional diversity character-taxon matrix and references. (XLSX)

S1 Fig Humeri of *Tethydraco* and *Pteranodon*. *T*. *regalis* FSAC-OB 1 (A) compared to *Pteranodon* (YPM 2709) (B). Arrows denote the position of the base of the deltopectoral crest and the ulnar crest.
(JPG)

S2 Fig Distal humerus of *Tethydraco* and *Pteranodon*. Comparison of *Pteranodon* YPM 1175 in ventral (A) and dorsal (C) views to *T*. *regalis* FSAC-OB 1 in ventral (B) and dorsal (D) views, showing the different degree of development of the entepicondyle and ectepicondyle.
(JPG)

S3 Fig Ulnae of *Tethydraco* and *Pteranodon*. (A) *T*. *regalis* FSAC-OB 199 and *Pteranodon* (B) YPM 2499, (C) YPM 2497, and (D) YPM 2409.
(JPG)

S4 Fig Femora of *Tethydraco* and *Pteranodon*. Femora of (A) *T*. *regalis* FSAC 201 and *Pteranodon* (B) YPM 2597 and (C) YPM 1175.
(JPG)

S5 Fig Variation in the humeri of *Alcione*. (JPG)

S6 Fig Humeri of *Alcione*, *Simurghia*, and *Barbaridactylus*. (A) *A*. *elainus* FSAC-OB 5 to (B) *S*. *robusta* FSAC-OB 7, and (C) *B*. *grandis* FSAC-OB 8.
(TIF)

S7 Fig Vectorized version of Fig 20. (EPS)

S1 Table Catalogue of specimens examined in the course of this study. (CSV)

S1 Text Notes on provenance and stratigraphy of the phosphate pterosaurs; systematics and taxonomy; discussion of affinities of *P*. *barbarulna*; and age of “*N*.*” lamegoi*. (DOCX)

## Abbreviations

FSAC: Faculté des Sciences Aïn Chock, Casablanca, Morocco
OCP: Office Cherifién des Phosphates, Khouribga, Morocco
PCA: principal component analysis
PCoA: principal coordinates analysis
YPM: Yale Peabody Museum, New Haven, United States
