# Correction: Late Maastrichtian pterosaurs from North Africa and mass extinction of Pterosauria at the Cretaceous-Paleogene boundary

**DOI:** 10.1371/journal.pbio.1002627

**Published:** 2018-04-11

**Authors:** Nicholas R. Longrich, David M. Martill, Brian Andres

## Notice of republication

This article was republished on March 28 2018, to correct the following figure errors that were introduced during the final publication preparation stages:

Fig 1B contained a satellite image that was not available for publication under the Creative Commons Attribution License (creativecommons.org/licenses/by/4.0/). Fig 1B has therefore been replaced with an image created by the author. The figure legend for Fig 1 has been updated to reflect this change.

Fig 6C, Fig 11C, Fig 11E and Fig 11F contained an incorrect scale bar. This has been corrected.

Fig 20 contained a typographical error which has been corrected.

Please download this article again to view the correct versions of the figures.
