# Supplementary figures and images for: Late Maastrichtian pterosaurs from North Africa and mass extinction of Pterosauria at the Cretaceous-Paleogene boundary
Source: PLoS Biol. 2018 Mar 13;16(3):e2001663. doi: 10.1371/journal.pbio.2001663 (PMC5849296; doi:10.1371/journal.pbio.2001663)

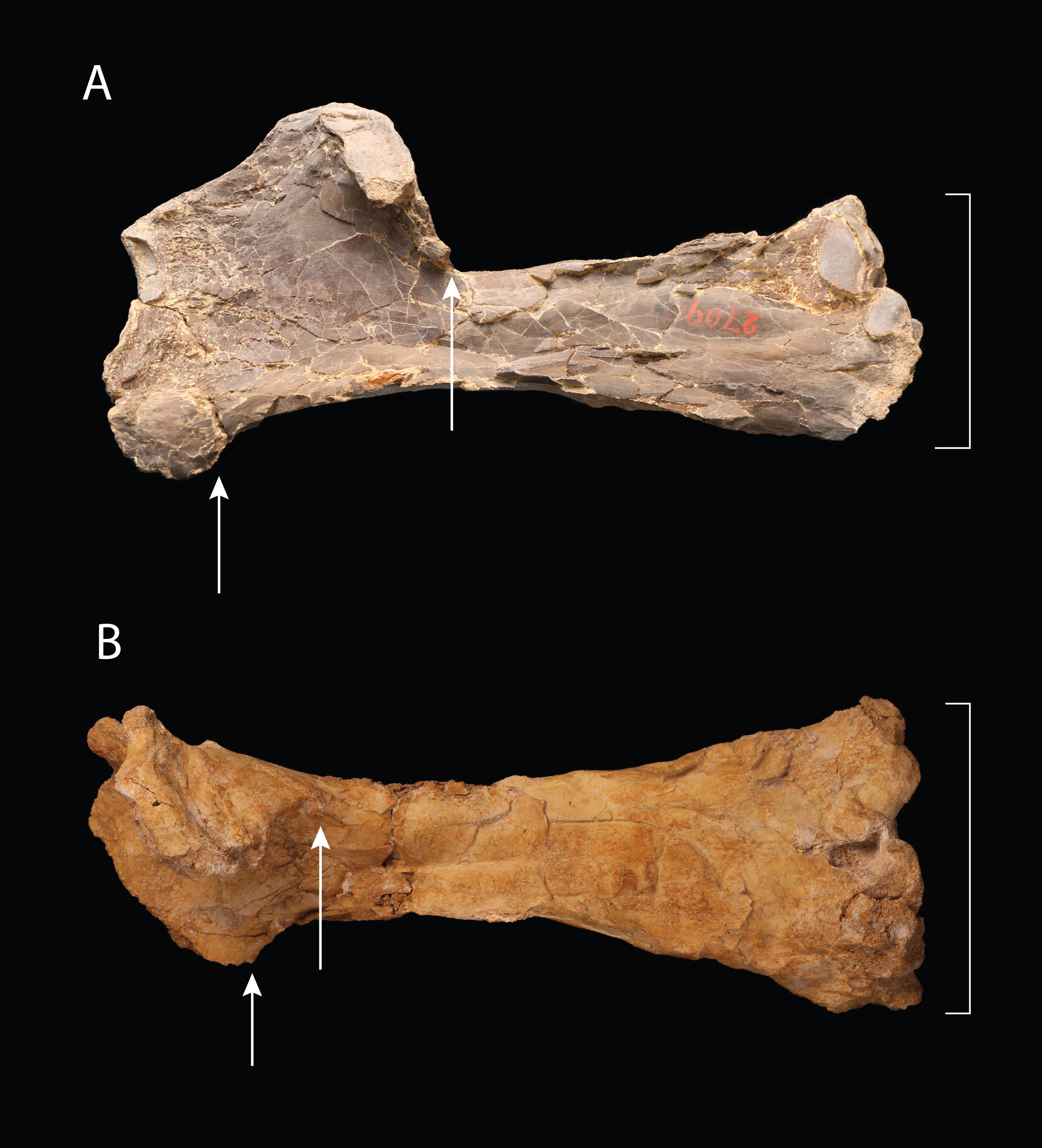

Supplement: S1 Fig — T. regalis FSAC-OB 1 (A) compared to Pteranodon (YPM 2709) (B). Arrows denote the position of the base of the deltopectoral crest and the ulnar crest. (JPG) [file pbio.2001663.s003.jpg]

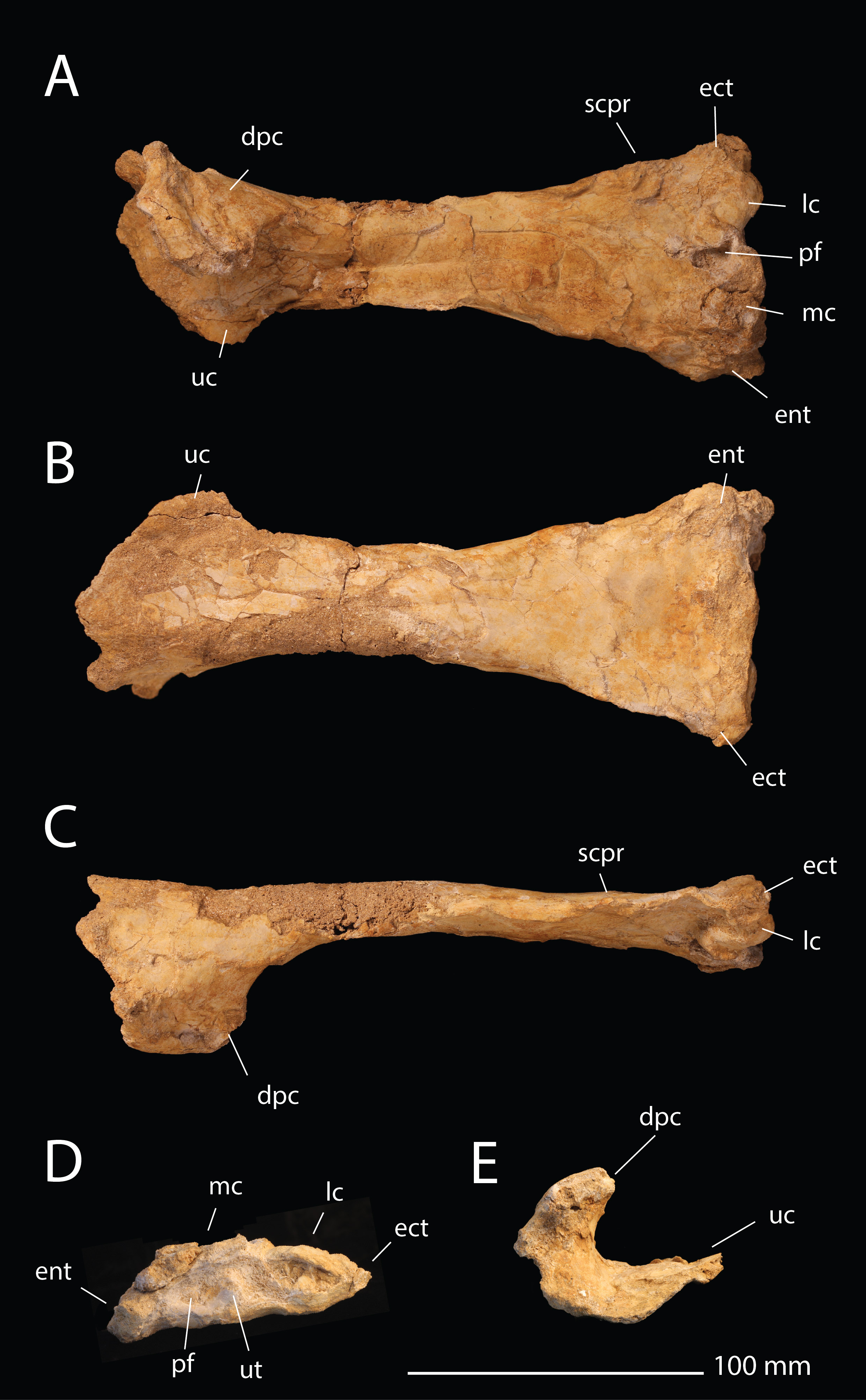

Supplement: S2 Fig — Comparison of Pteranodon YPM 1175 in ventral (A) and dorsal (C) views to T. regalis FSAC-OB 1 in ventral (B) and dorsal (D) views, showing the different degree of development of the entepicondyle and ectepicondyle. (JPG) [file pbio.2001663.s004.jpg]

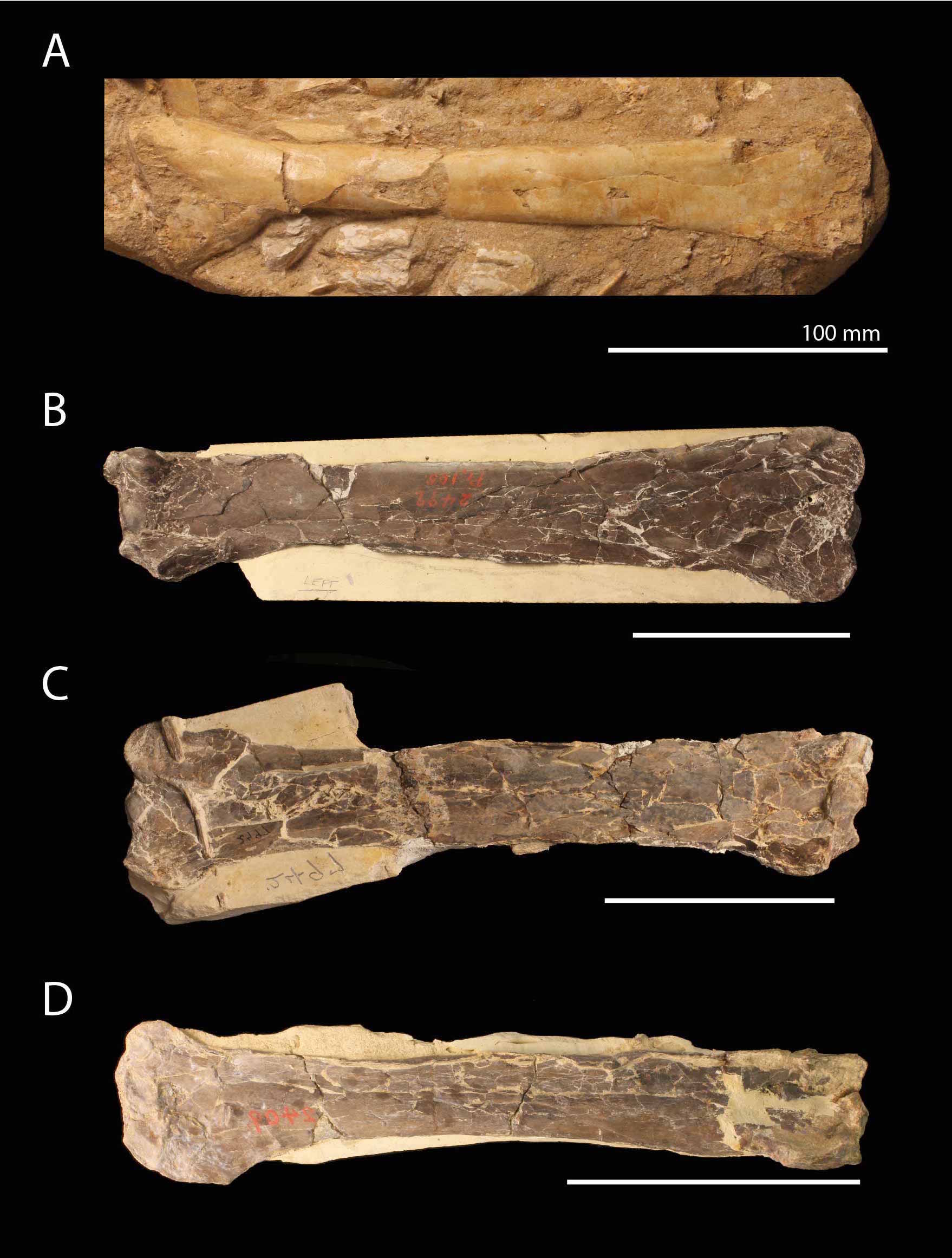

Supplement: S3 Fig — (A) T. regalis FSAC-OB 199 and Pteranodon (B) YPM 2499, (C) YPM 2497, and (D) YPM 2409. (JPG) [file pbio.2001663.s005.jpg]

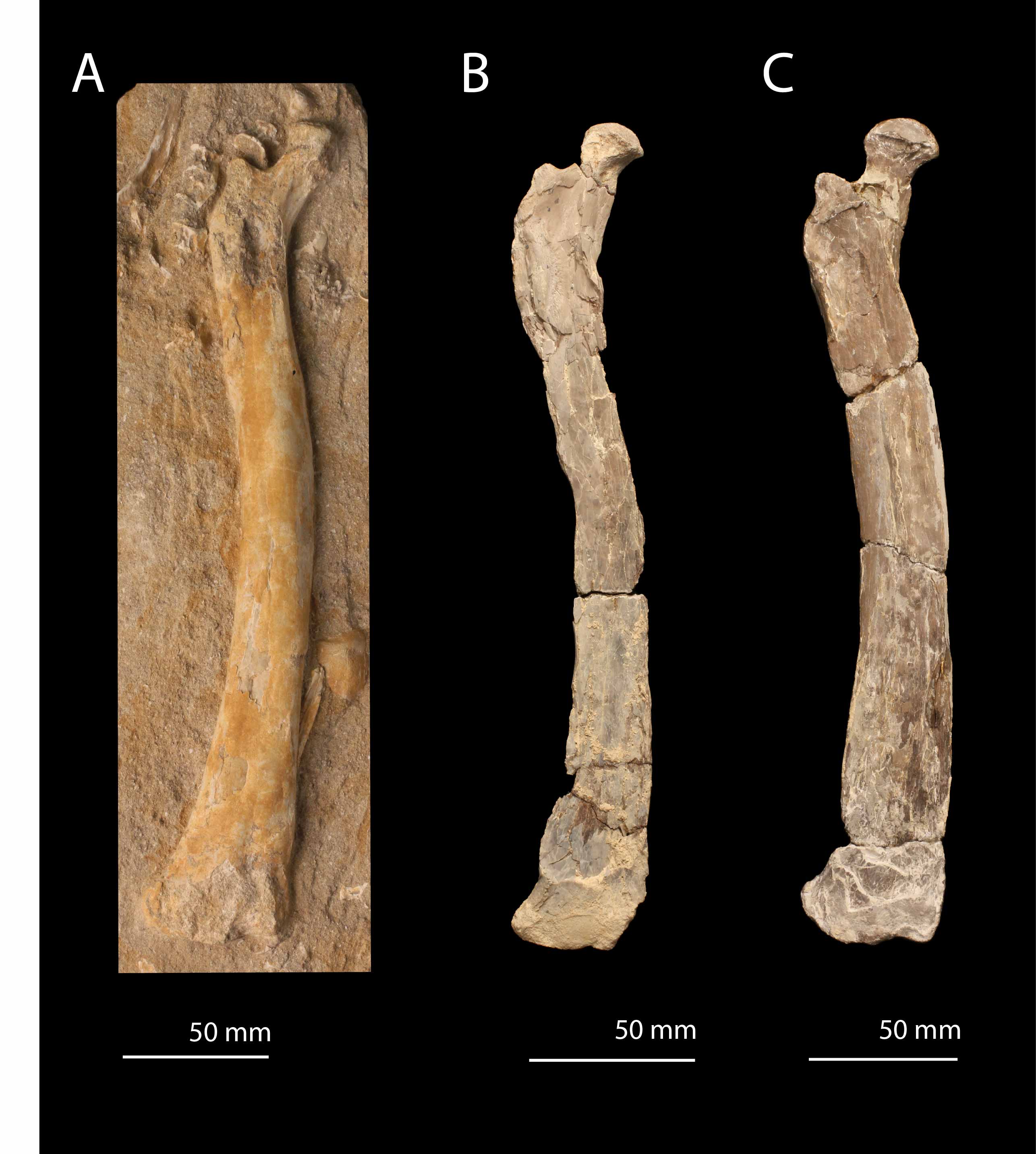

Supplement: S4 Fig — Femora of (A) T. regalis FSAC 201 and Pteranodon (B) YPM 2597 and (C) YPM 1175. (JPG) [file pbio.2001663.s006.jpg]

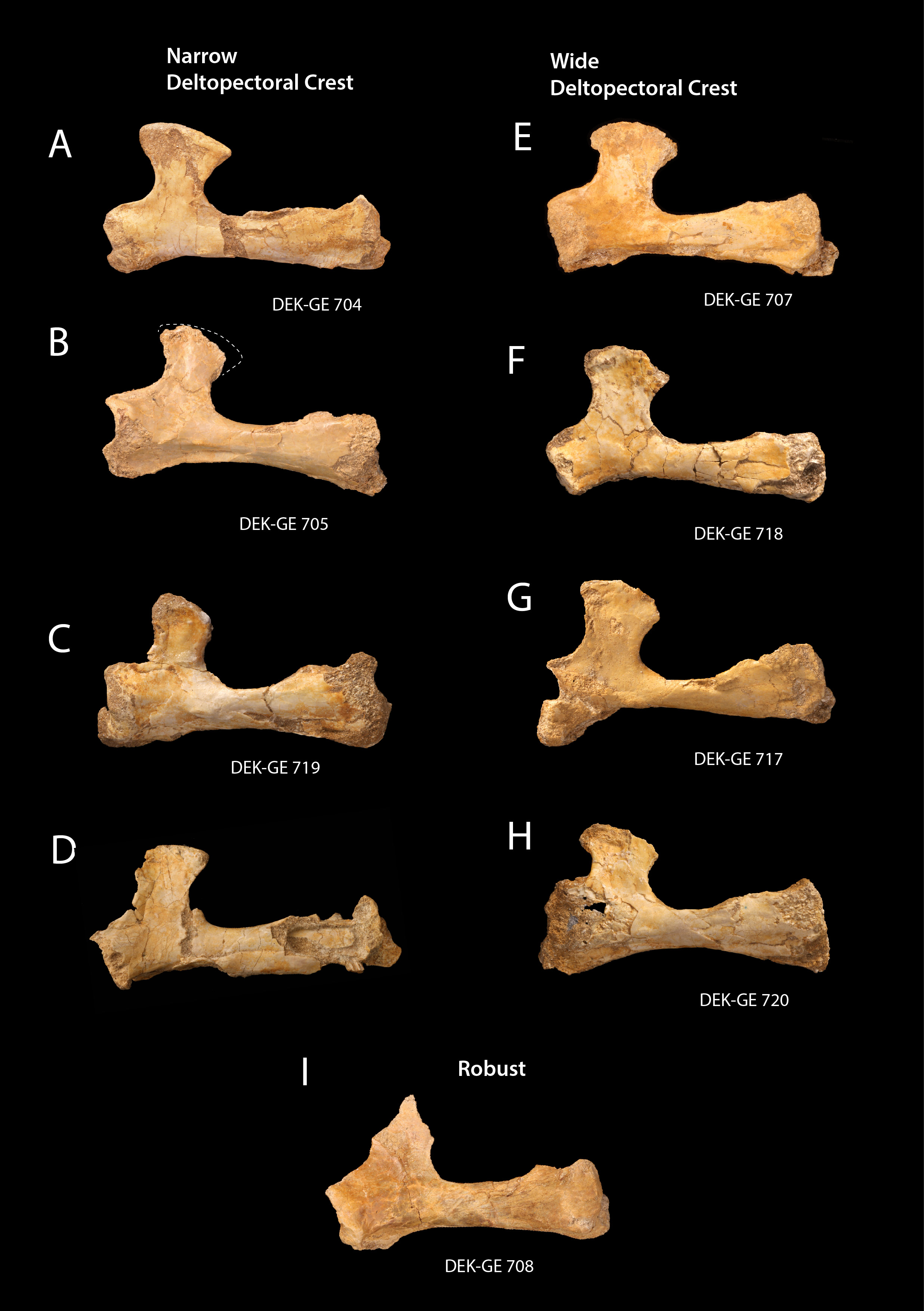

Supplement: S5 Fig — (JPG) [file pbio.2001663.s007.jpg]

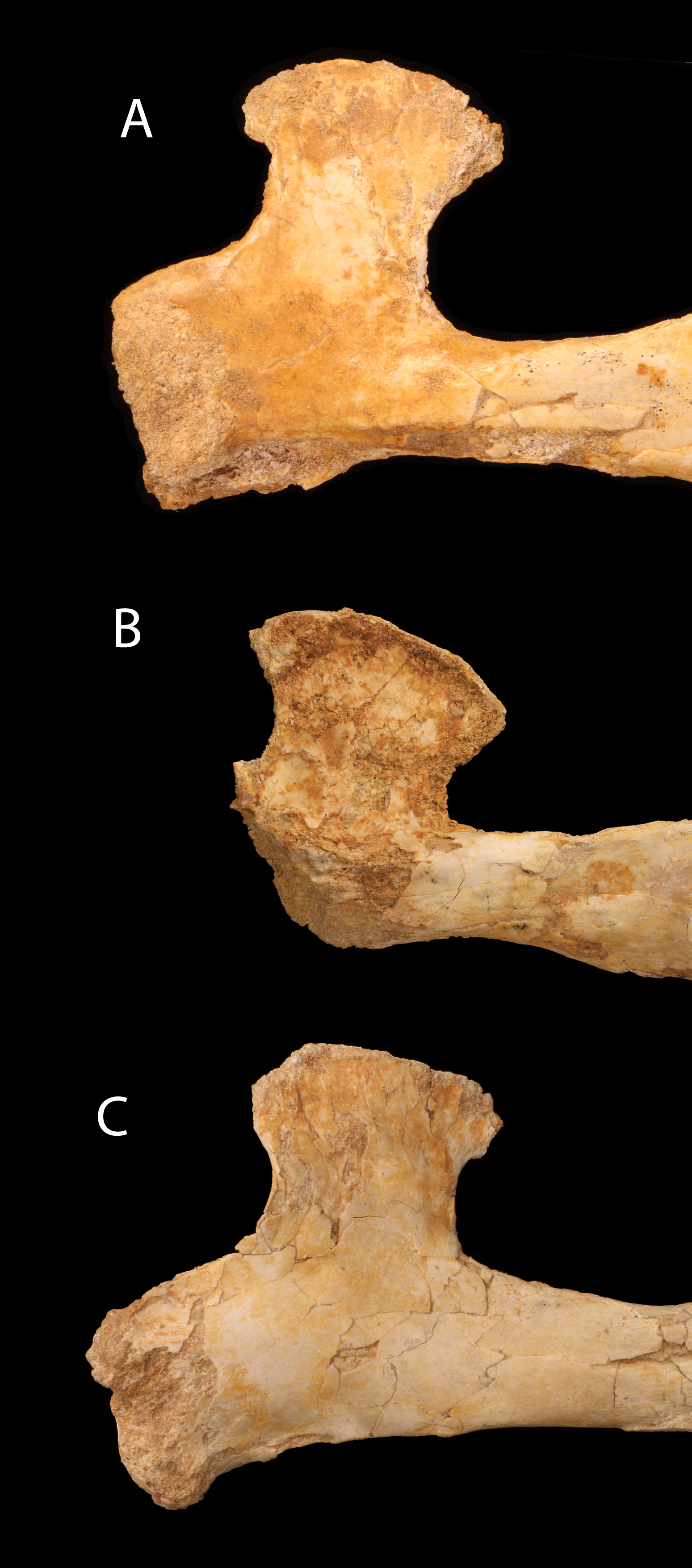

Supplement: S6 Fig — (A) A. elainus FSAC-OB 5 to (B) S. robusta FSAC-OB 7, and (C) B. grandis FSAC-OB 8. (TIF) [file pbio.2001663.s008.tif]
