# Supplementary material for: Late Maastrichtian pterosaurs from North Africa and mass extinction of Pterosauria at the Cretaceous-Paleogene boundary
Source: PLoS Biol. 2018 Mar 13;16(3):e2001663. doi: 10.1371/journal.pbio.2001663 (PMC5849296; doi:10.1371/journal.pbio.2001663)
Supplement: S1 Text — (DOCX) [file pbio.2001663.s011.docx]

**Supplementary Information 1**

**Table of Contents**

1. **Provenance and Stratigraphy**
2. **Systematics and Taxonomy**
3. **Affinities of *Piksi barbarulna***
4. **Age of “*Nyctosaurus*” *lamegoi***
5. **References**

**1. Provenance and Stratigraphic Constraint**

All fossils are from the late Maastrichtian (‘Couche III’) phosphates of the Ouled Abdoun basin of Morocco, southeast of Casablanca. Provenance data was obtained from local fossil merchants and verified by inspection of the matrix. Couche III matrix consists of a mixture of sand sized phosphate pellets and fragments of bone. The bulk of the material comes from the middle/lower Couche III beds near Sidi Daoui, in a regionally extensive bonebed with abundant vertebrate bones and teeth. This bed exhibits a distinctive mottled orange-and-blue-grey matrix, and bones tend to have a yellow-orange tinge. A minority of material is from the Couche III beds at Sidi Chennane, which are of similar age; Couche III bones tend to be paler in color and the matrix is typically grey. This combination of features- the abundant bone clasts, abundant shark and mosasaur teeth, and the distinctive coloration of the matrix and fossils- make it possible to place the fossils into stratigraphic context.

Correlation of the Couche III beds is complicated by the fact that they lack shelly fossils or microfossils such as ammonites and foraminifera. Neither do the phosphates preserve organic fossils such as fossil pollen or dinoflagellates. Accordingly, a Late Maastrichtian age was previously established on the basis of vertebrate remains, specifically sharks (Noubhani and Cappetta, 1997). Other elements of the fauna such as the mosasaurs *Mosasaurus* (Bardet et al., 2004) and *Carinodens* (Schulp et al., 2009), which are known from the Maastrichtian of Europe, are consistent with this assessment.

More recently, the carbon and oxygen isotopes of biogenic apatite were studied to correlate the phosphates to the global carbon and oxygen curves (Yans et al., 2014). Based on this work, the Couche III beds of Sidi Daoui can be correlated to the latest Maastrichtian, no more than 1.5 Ma before the K-Pg boundary, perhaps less. The lower Couche III beds contain a shark fauna that is identical to that of the upper beds (C. Underwood pers. comm. 2016) suggesting that this fauna is not markedly older.

Some pterosaur does appear come from an underlying bed, which locals have dubbed ‘Couche IV’. Couche IV is reported as occurring about 2 meters below Couche III. Couche IV sediments are readily recognized by their pale grey color, chalky consistency, and bleached white bones. Preliminary study of Couche IV suggests that it contains a distinct mosasaur fauna, suggesting that it is older than Couche III. However, none of the material included in this paper comes from Couche IV.

**2. Systematics and Taxonomy**

We chose the humerus as the basis for our taxonomy because it is diagnostic, abundant in the assemblage, and consistently preserved in a wide range of Late Cretaceous taxa.

The humerus exhibits numerous potentially diagnostic characters including the proportions of the humeral shaft, the size and shape of the deltopectoral crest, the morphology of the distal condyles, the position and shape of the ectepicondyle and entepicondyle, and the existence of pneumatic fossae and foramina. In combination, these characters make it possible to diagnose taxa. Furthermore, there is an established precedent of diagnosing and naming taxa based on the humerus (e.g. *Bennetazhia oregonensis*, *Nyctosaurus lamegoi*).

Based on the humerus, at least four distinct genera- *Tethydraco*, *Barbaridactylus*, *Alcione*, and *Simurghia*- can be diagnosed. Within *Alcione*, there is extensive variation in the size and shape of the deltopectoral crest and the ectepicondyle and entepicondyle, suggesting that more than one species may be represented.

The utility of this approach has been validated by subsequent discoveries. *Alcione* was initially diagnosed based on an isolated humerus, and subsequently an associated skeleton (the holotype) was identified, corroborating its identification as a distinct taxon. Similarly, *Barbaridactylus* was originally diagnosed based on an incomplete humerus, and more complete remains eventually came to light, corroborating its recognition as a distinct taxon; identification of the humerus of *Tethydraco* as pteranodontid was followed by the discovery of pteranodontid ulnae and hindlimb material.

**3. Affinities of *Piksi barbarulna***

*Piksi* is a highly controversial taxa. Originally identified as an ornithurine bird by Varricchio (Varricchio, 2002) it was subsequently reclassified as a pterosaur by Agnolin and Varricchio (Agnolin and Varricchio, 2012). A more recent study claimed that *Piksi* represents a non-avian theropod (Martin-Silverstone et al., 2016). No consensus currently exists among pterosaur workers.

*Piksi* would clearly be unusual among pterosaurs in terms of the morphology of the humeral distal condyles, the morphology of the olecranon fossa and its small size. Neither does it closely resemble any known pterosaur lineage. However, comparisons with birds, theropods and pterosaurs show that *Piksi* exhibits few if any derived characters of birds or theropods that are not also seen in pterosaurs. Instead, *Piksi* exhibits a series of derived characters that are found in pterosaurs.

Characters supporting referral to Pterosauria or a more exclusive clade inside Pterosauria include:

1. **Division of the lateral condyle of the humerus by a trochlea into two distinct articular surfaces.** In Pteranodontoidea (Hooley, 1913; Averianov et al., 2005) and Azhdarchoidea (Averianov, 2010), the lateral condyle (capitulum) is divided by a groove into a distinct medial and lateral facet, typically a large medial articular surface and a smaller, lateral, accessory articular surface. This same feature is seen in *Piksi* (Agnolin and Varricchio, 2012), with this accessory articular surface misidentified as the “ectepicondyle”. The bone here lacks rugosities or pits for muscle insertion however and is instead smooth and inflated as in the articular surfaces, indicating that it is part of a joint. Similarly the structure identified as the “ectepicondyle” in some other pterosaurs (Averianov et al., 2005) is the accessory articular surface of this divided lateral condyle.
2. **Pneumatic foramen between the articular condyles of the humerus.** A well-developed pneumatic foramen is present between the medial and lateral condyles, as in pteranodontoids, nyctosaurids (NRL pers obs. of *Alcione*) and perhaps azhdarchids (NRL pers. obs. of Kem Kem azhdarchid humerus). An ovoid, matrix-filled opening representing this pneumatic foramen is present between the medial and lateral condyles in *Piksi* (Agnolin and Varricchio, 2012).
3. **Deep olecranon fossa on the distal end of the humerus, behind the condyles.** As in azhdarchoids (Averianov, 2010) and pteranodontoids (Hooley, 1913; Averianov et al., 2005) the distal end of the humerus bears a deep depression posterior to the condyles, the olecranon fossa. This fossa is seen in *Piksi*, although it is shifted onto the posterior aspect of the humerus, and unusually deep. In pterosaurs, this fossa typically bears a pneumatic opening. This pneumatic opening could be present in *Piksi*, represented by a small matrix-filled space behind the medial condyle, but preparation or scanning would be required to verify the presence of this feature..
4. **Strong expansion of the proximal articular surface of the radius.** The head of the radius is weakly expanded in birds and theropods. In pterosaurs, it is strongly expanded in azhdarchids (Averianov, 2010) and in pteranodontoids (Eaton, 1910; Hooley, 1913; Bramwell and Whitfield, 1974), with head being better developed laterally than medially. The same condition is seen in *Piksi* (Agnolin and Varricchio, 2012).
5. **Strong expansion and flattening of the proximal end of the ulna.** In contrast to birds, theropods, or the basal archosaur condition seen in crocodylomorphs, the head of the ulna is expanded and compressed in azhdarchoids (Averianov et al., 2005) and pteranodontoids }(Hooley, 1913). The proximal end of the ulna is strongly compressed in *Piksi* (Agnolin and Varricchio, 2012), as in pterosaurs.
6. **Shaft of ulna deflected relative to humerus.** In pterosaurs, the proximal end of the ulna is angled relative to the shaft, such that the ulna is deflected with respect to the humeral shaft when the antebrachium is fully extended. This condition is seen in both azhdarchoids (Averianov, 2010) and pteranodontoids (Eaton, 1910; Hooley, 1913). The same condition is seen in *Piksi*.
7. **Distal end of the ulna flattened.** The distal end of the ulna is flattened in pterosaurs including both azhdarchoids (Averianov, 2010) and pteranodontoids (Hooley, 1913).
8. **Distal end of ulna bearing a tubercle.** The ulna bears a tubercle on its distal end in both azhdarchoids (Averianov, 2010) and pteranodontoids (Hooley, 1913). *Piksi* shares this same feature (Agnolin and Varricchio, 2012).

The following features are potentially informative with respect to *Piksi*’s placement in Pterosauria:

1. **Distal end of humerus triangular.** In azhdarchoids, the humerus projects behind the olecranon fossa to create a rounded, D-shaped profile to the distal humerus. This feature is absent in *Piksi*, instead the back of the humerus is concave, more similar to the triangular distal end of the humerus seen in pteranodontoids including ornithocheirans (Kellner and Tomida, 2000) and istiodactylids (Hooley, 1913).
2. **Hypertrophied accessory articular surface of medial condyle.** In most pterosaurs, such as Azhdarchidae (Averianov, 2010), the medial condyle has a small accessory surface that is separated by a groove from the main body of the condyle. In *Piksi* (Agnolin and Varricchio, 2012) this accessory surface is greatly elaborated. A similar elaboration of the accessory surface is seen in a humeral fragment from the Volgograd region of Russia (Averianov et al., 2005) and in Istiodactylus (Hooley, 1913).
3. **Proximal end of ulna divided by a shallow notch.** The proximal end of the humerus in *Piksi* has a concave margin where the olecranon process should be in a bird or a theropod. This feature is absent in Azhdarchidae (Averianov et al., 2005) and the pterodactyloid *Alcione* but a similar shallow notch and divided ulna head is seen in *Istiodactylus* (Hooley, 1913).

The character evidence is therefore in agreement with the interpretation of *Piksi* as a member of Pterosauria, and specifically suggests affinities with Pteranodontoidea.

**4. Age of “*Nyctosaurus*” *lamegoi***

The existence of nyctosaurids in the Maastrichtian has been widely (Wellnhofer, 1991; Witton, 2013; Benson et al., 2014) but not universally (Unwin, 2005) accepted on the basis of “*Nyctosaurus*” *lamegoi* (Wellnhofer, 1991; Benson et al., 2014) an isolated humerus from the Late Cretaceous of Brazil (Price, 1953). Our analysis follows previous studies (Benson et al., 2014) in supporting nyctosaurid affinities for this taxon. However, the age of the specimen is uncertain; as discussed below the primary literature makes it clear that the specimen has poor stratigraphic constraint.

Although the specimen has been repeatedly reported as coming from the Maastrichtian {Wellnhofer, 1991 #150}, with the with range of nyctosaurids typically shown extending up to the K-Pg boundary {Benson, 2014 #3480}, the situation is more complicated. Price (Price, 1953) reported the specimen as from a collection made by Colonel João Domingues dos Santos from the Fazenda do Congo farm in Paraiba State; the fossils came from the right bank of the Rio Gramame (Price, 1953). The collection contains fossils from two distinct faunas. One is Maastrichtian in age, and pertains to the Gramame Formation. The second is Campanian in age, and comes from the underlying Itamaraca Formation (Price, 1953). Thus, the specimen could be either Campanian or Maastrichtian in age (Price, 1953).

Due to the lack of detailed provenance data and the fact that both Campanian and Maastrichtian fossils occur in the collection, it is difficult to definitely assign an age to “*Nyctosaurus*” *lameghoi*. Examinations of associated matrix suggest that the specimen comes from the Gramame Formation and that it was “probably Maastrichtian” (Price, 1953), but this assignment was made “with reservations” (Price, 1953) because in places the sediments of the formations can be difficult to distinguish. As a result, it is likely- but by no means certain- that the specimen is Maastrichtian.

However, even assuming that the specimen does come from the Maastrichtian, however, the Maastrichtian spans some 6 million years of time (Gradstein et al., 2012); the specimen could concievably come from well below the K-Pg boundary. As a result, regardless of which formation the specimen comes from, the specimens described from the phosphates are the only ones that can be confidently identified as coming from the late Maastrichtian.

**5. References**

Agnolin, F.L., Varricchio, D., 2012. Systematic reinterpretation of *Piksi barbarulna* Varricchio, 2002 from the Two Medicine Formation (Upper Cretaceous) of Western USA (Montana) as a pterosaur rather than a bird. Geodiversitas 34, 883-894.

Averianov, A., 2010. The osteology of *Azhdarcho lancicollis* Nessov, 1984 (Pterosauria, Azhdarchidae) from the late Cretaceous of Uzbekistan. Proceedings of the Zoological Institute RAS 314, 264-317.

Averianov, A.O., Kurochkin, E.N., Pervushov, E.M., Ivanov, A.V., 2005. Two bone fragments of ornithocheiroid pterosaurs from the Cenomanian of Volgograd Region, southern Russia. Acta Palaeontologica Polonica 50.

Bardet, N., Suberbiola, X.P., Iarochene, M., Bouyahyaoui, F., Bouya, B., Amaghzaz, M., 2004. Mosasaurus beaugei Arambourg, 1952 (Squamata, Mosasauridae) from the late Cretaceous phosphates of Morocco. Geobios 37, 315-324.

Benson, R.B., Frigot, R.A., Goswami, A., Andres, B., Butler, R.J., 2014. Competition and constraint drove Cope's rule in the evolution of giant flying reptiles. Nature Communications 5.

Bramwell, C., Whitfield, G.R., 1974. Biomechanics of *Pteranodon*. Philosophical Transactions of the Royal Society B 267, 503-581.

Eaton, C.F., 1910. Osteology of *Pteranodon*. Memoirs of the Connecticut Academy of Arts and Sciences 2, 1-38.

Gradstein, F.M., Ogg, G., Schmitz, M. 2012. The Geologic Time Scale 2012. Elsevier, 2.

Hooley, R.W., 1913. On the skeleton of *Ornithodesmus latidens*; an ornithosaur from the Wealden Shales of Atherfield (Isle of Wight). Quarterly Journal of the Geological Society 69, 372-422.

Kellner, A.W.A., Tomida, Y., 2000. Description of a new species of Anhangueridae (Pterodactyloidea) with comments on the pterosaur fauna from the Santana Formation (Aptian-Albian), northeastern Brazil. National Science Museum Monographs 17, ix-137.

Martin-Silverstone, E., Witton, M.P., Arbour, V.M., Currie, P.J., 2016. A small azhdarchoid pterosaur from the latest Cretaceous, the age of flying giants. Royal Society Open Science 3, 160333.

Noubhani, A., Cappetta, H. 1997. Les Orectolobiformes, Carcharhiniformes et Myliobatiformes (Elasmobranchii, Neoselachii) des Bassins à phosphate du Maroc (Maastrichtien-Lutétien basal): systématique, biostratigraphie, évolution et dynamique des faunes. Pfeil.

Price, L.I., 1953. A presença de Pterosauria no Cretáceo Superior do Estado da Paraíba. Notas Preliminares e Estudos, Divisao de Geologia e Mineralogia, Brasil 71, 1-10.

Schulp, A.S., Bardet, N., Bouya, B., 2009. A new species of the durophagous mosasaur *Carinodens* (Squamata, Mosasauridae) and additional material of *Carinodens belgicus* from the Maastrichtian phosphates of Morocco. Netherlands Journal of Geosciences 88, 161-167.

Unwin, D.M. 2005. The pterosaurs from deep time. Pi Press, New York.

Varricchio, D.J., 2002. A new bird from the Upper Cretaceous Two Medicine Formation of Montana. Canadian Journal of Earth Sciences 39, 19-26.

Wellnhofer, P. 1991. The Illustrated Encyclopedia of Pterosaurs. Salamander Books, Hong Kong 192 pp.

Witton, M.P. 2013. Pterosaurs: natural history, evolution, anatomy. Princeton University Press.

Yans, J., Amaghzaz, M.B., Bouya, B., Cappetta, H., Iacumin, P., Kocsis, L., Mouflih, M., Selloum, O., Sen, S., Storme, J.-Y., 2014. First carbon isotope chemostratigraphy of the Ouled Abdoun phosphate Basin, Morocco; implications for dating and evolution of earliest African placental mammals. Gondwana Research 25, 257-269.
